# ANEMI3: An updated tool for global change analysis

**DOI:** 10.1371/journal.pone.0251489

**Published:** 2021-05-10

**Authors:** Patrick A. Breach, Slobodan P. Simonovic

**Affiliations:** Department of Civil and Environmental Engineering, Western University, London, Canada; Universidade de Vigo, SPAIN

## Abstract

The ANEMI model is an integrated assessment model of global change that emphasizes the role of water resources. The model is based on the principles of system dynamics simulation to analyze changes in the Earth system using feedback processes. Securing water resources for the future is a key issue of global change, and ties into global systems of population growth, climate change, carbon cycle, hydrologic cycle, economy, energy production, land use and pollution generation. Here the third iteration of the model–ANEMI3 is described, along with the methods used for parameter estimation and model testing. The main differences between ANEMI3 and previous versions include: (i) implementation of the energy-economy system based on the principles of system dynamics simulation; (ii) incorporation of water supply as an additional sector in the global economy that parallels the production of energy; (iii) inclusion of climate change effects on land yield and potentially arable land for food production, and (iv) addition of nitrogen and phosphorus based nutrient cycles as indicators of global water quality, which affect the development of surface water supplies. The model is intended for analyzing long-term global feedbacks which drive global change. Because of this, there are limitations related to the spatial scale that is used. However, the model’s simplicity can be considered a strength, as it allows for the driving feedbacks to be more easily identified. The model in its current form allows for a variety of scenarios to be created to address global issues such as climate change from an integrated perspective, or to examine the change in one model sector on Earth system behaviour. The endogenous structure of the model allows for global change to be driven entirely by model structure rather than exogenous inputs. The new additions to the ANEMI3 model are found to capture long term trends associated with global change, while allowing for the development of water supplies to be represented using an integrated approach considering global economy and surface water quality.

## Introduction

Human impacts on the environment at global scales are being realized through our ability to alter atmospheric concentrations of greenhouse gases and consequently global climate, creating the need to consider environmental problems and their interactions with the Earth as a system. The Earth system is composed of biological, physical, chemical, and human elements that form a network of feedbacks through their interconnections [1]. The concept of global change becomes increasingly important as the Earth system components such as population, economic productivity, climate, food production, and hydrology are interlinked through dynamic non-linear feedback processes [2]. Within this system, changes in one component inevitably lead to changes in another. Therefore, global change research focuses on interactions between components of the Earth system, compared to only those of climate [1, 3].

Assessment of various aspects of global change often requires the use of models from different domains and a way to combine them so that the relationships and interactions between these models can be studied. When it comes to global change research, the goal is often to analyze the effect of policies or scenarios on different aspects of global change to help inform decision makers. This has necessitated the use of new tools and modelling paradigms to analyze complex interactions within the Earth system at a variety of spatial and temporal scales.

The integrated assessment modelling (IAM) approach involves the coupling of disciplinary models. This is done by exchanging inputs and outputs that would otherwise be exogenous to each model. Connections can be made in one direction (from one disciplinary model to another) or in both directions, creating a feedback loop between the two models. Due to the increased complexity in the combined model, simplified forms of the disciplinary models are often used. Among many examples, Holden and Edwards [4] present an approach on how to reduce the complexity of an atmosphere-ocean global climate model to incorporate it into an integrated assessment model of global change [4].

The principles of system dynamics simulation provide an ideal framework for the development of integrated assessment models. System dynamics simulation is driven purely by feedback and delay processes that allow for systems to evolve over time from an initial simulation point. The structure of system dynamics models relies on the configuration of stocks or state variables and flows or rate variables. Flows act to alter the stocks, while any number of auxiliary variables can be used to define the flows further. These basic building blocks can be arranged to represent any number of system structures resulting in different behaviours. Integrated assessment models can be developed within this framework by representing the disciplinary models as sub-systems or sectors. Each sector may be driven by feedback processes and is connected to other sectors through intersectoral feedbacks. The ANEMI3 model is built using these principles and extends previous versions (ANEMI1 and ANEMI2) to incorporate additional intersectoral feedbacks, providing a more complete picture of the Earth system. In addition, a major rework of the energy-economy sector has allowed for dynamics of water supply development to be incorporated alongside energy production. In this model, the development of water supplies both conventional (surface water and groundwater) and alternative (desalination and water reuse) are included within the Earth system using an integrated, feedback-based approach.

The main objectives of this paper are to i) document the structure of the ANEMI3 model, ii) identify feedback processes that arise from the structure, and iii) propose ways in which the model can be utilized. The following sections provide a review of integrated assessment models and system dynamics in the literature, a description of the model sectors and feedbacks, information on parameter estimation and testing of the model, and limitations of ANEMI3.

## Literature review

### Integrated assessment modelling

Many different methods can be used to form a model for integrated assessment. Connections between disciplinary models can be made statically (output of one model is first obtained then given as input to another), or dynamically (both models running at the same time). The latter of which is the only way that feedback loops can be created and studied. Dynamic connections can be made by using a computer program to facilitate the exchange of information while the models are running, or both models can be combined into the same computer code [5]. The field of system dynamics focuses specifically on analyzing the dynamic nature of systems composed of feedback loops. Therefore, system dynamics is ideal for constructing integrated assessment models of global change.

The representation of water resources in integrated assessments of global change varies widely in the amount of detail included and the level of integration with other components of the Earth system. In addition, the spatial scales at which components are represented and interact, vary in each IAM. The concept of dealing with mismatching spatial scales in integrated assessment research has been recognized as one of the major challenges. It defines how regional to local scale processes interact with the global system [6]. For example, in water resources management, this effect could be manifested through impacts on the global economy as agricultural and industrial production is limited by regional water stress.

However, as IAMs continue to evolve, there becomes a tighter integration between these sectors and biogeophysical cycles of the Earth system [7]. This integration has led to more comprehensive representations of the hydrologic cycle to assess impacts on water stress through comparisons of water availability and demand [8]. The models that currently integrate water availability and demand are: AIM (Asia-Pacific Integrate Model) [9], IMAGE (Integrated Model to Assess the Global Environment) [10], IGSM-WRS, (Integrated Global System Model which includes a Water Resource System component) [8], GCAM (Global Change Assessment Model) [11], and ANEMI [2, 12]. A sectoral comparison of these IAMs and other prevalent IAMs in the literature allows for examination of the focus area in each (Table 1). The model sectors in each IAM shown are incorporated in different ways. Here we define each sector as being either endogenous (dynamic, two-way connection with the rest of the model), exogenous (one-way connection either to or from the model), or not applicable (sector is not present). This comparison shows that all models have climate, energy, and emissions sectors in common. This is due to the issue of climate change being one of the first issues to be assessed from an integrated perspective at the global scale.

**Table 1:** Comparison of model sectors present among IAMs in the literature.

	MESSAGE	AIM	GCAM	IMAGE	DICE/RICE	FREE	IGSM-WRS	ANEMI3
**Agriculture**	x	x	x	x	x	---	x	x
**Land Use**	x	x	x	x	o	o	x	x
**Demography**	o	o	o	o	o	o	x	x
**Climate**	x	x	x	x	x	x	x	x
**Water Quality**	---	x	---	x	---	---	x	x
**Water Availability**	---	x	x	x	---	---	x	x
**Water Demand**	x	x	x	x	---	---	x	x
**Water Supply**	---	---	x	o	---	---	o	x
**Energy**	x	x	x	x	x	x	x	x
**Sea Level Rise**	---	x	x	x	---	---	x	x
**Economy**	x	o	x	o	x	x	x	x
**Emissions**	x	x	x	x	x	x	x	x

x = Endogenous, o = Exogenous, — = N/A (Not applicable).

In many cases, population dynamics or demography is treated as an exogenous component by incorporating external population projections as an input to the IAM. The most variation among IAMs lies in the water-related sectors, as not one sector is common across models. The ANEMI3 model is shown to be the only one with endogenous representations of water quality, availability, demand, and supply.

The AIM and IMAGE models are similar in that the Earth system is driven by a set of exogenous socioeconomic scenarios which are defined by trajectories of future population, GDP, and technological trends. These inputs drive feedback processes between land use, energy supply and demand, and CO_2_ emissions, which are used to assess regional impacts on water resources such as flood risk and water stress among other impacts on biodiversity, agricultural productivity, and ecosystems. The way these models are driven exogenously through future assumptions in the social-economy do not allow for cross-sectoral feedbacks between water resources systems and the dynamic evolution of global change. A combination of regional and grid based spatial disaggregation methods are used to resolve impacts of global change on finer spatial scales, however, in both models, there is no ability for impacts at this scale to feedback into the global system.

IGSM-WRS is a modified version of IGSM, which allows for the coupling of IGSM’s Earth system model to a detailed water resource system (WRS) [8]. This WRS calculates water availability from surface storage and groundwater resources within a set of 282 large river basins around the World, using a global hydrologic model on a 2x2.5-degree grid. Alternative water sources are also accounted for through water diversion from neighbouring grid cells and desalination capacity in coastal environments. Although the model includes a comprehensive water sector and allows for assessment at the regional scale in the context of the global system, it was noted that the water sector is related to the economy-climate sector via a “one-way” relationship [8].

The GCAM model has undergone recent updates that greatly improve water resources representation for assessments of global change [11]. A water resources system was added, representing water availability, supply, and demand at a basin level consisting of 235 sub-basins globally. Availability of water is simulated using a global hydrological model, while demands are based on gridded estimates of electricity production for industry, and irrigation demands for agriculture. Municipal demands are based on exogenous gridded values for population and GDP. Water supplies are represented by surface water and groundwater resources in the hydrologic model as well as seawater for desalination. The production of these supplies is dependent on the economics of each supply type in a given region. For example, desalination is used in places where surface water and groundwater are not abundant, and desalination is cheaper (i.e., closer to coastlines). Groundwater depletion and changes in surface water availability also affect water price allowing for the use of these supply options to change [13]. Feedback effects between model sectors include connections between energy, water, land, climate, and socioeconomics in GCAM version 5.1 [11]. The incorporation of water resources in GCAM is a major improvement, however representations of water quality and water reuse are currently not included. Furthermore, population is still an exogenous policy variable which limits potential negative feedback from resource constraints and pollution, such as water stress and water quality degradation.

### System dynamics simulation approach

A system can be generally defined as a collection of structural and non-structural elements that are connected and interact with each other to function as a whole [14, 15]. This definition encompasses many types of systems that could be physical, organizational, social, or abstract. A system typically has an input that generates an output through a series of transformations. In open systems, the output leaves the system boundary, while in closed systems the output goes on to affect the input, thus creating a feedback loop.

The study of system dynamics simulation seeks to find endogenous explanations of system behaviour [16]. What this means is the source of the problem being investigated lies within the system structure. Exogenous explanations of system behaviour do not explain the dynamics of the system responsible for the problem–they only pose further questions on what caused the exogenous variables to change as they did [16]. Endogenous system behaviour can be mapped out using causal loop diagramming in order to identify feedback relationships.

System dynamics simulation implements the principles of systems thinking to decompose real world problems into systems built of interconnected elements. Systems thinking facilitates the conceptualization of system dynamics simulation models through the formulation of dynamic hypotheses (how a system will behave over time). This process involves the use of causal loop diagramming to map out the feedback loops that are driving system behaviour. System dynamics simulation builds from the conceptual models developed through systems thinking by adding structure to them. The addition of stocks or state variables, and the flows that affect them take the system from a conceptual model to a mathematical model through stock and flow diagramming. Stock and flow diagrams illustrate the configuration of stocks and flows, which is essentially a visual representation of a system of first-order differential equations. Most, if not all, IAMs can be represented in this way from a high level. For these reasons, the system dynamics simulation approach is ideal for constructing IAMs and provides a formalized way for creating feedback loops between disciplinary models of global change.

Anthropogenic influence on the Earth system in the form of a growing population with increased usage of natural resources and pollution of the air, soil, and water is causing global changes in climate and the availability and quality of freshwater supplies. The use of alternative water supplies such as desalination and water reuse technologies provide a potential means to alleviate water stress. Improving the security of freshwater resources has been identified as one of the main objectives of prospective global change research, which is becoming increasingly integrated amongst various disciplines. Therefore, an integrated approach is needed to address research in this area. Integrated assessment modelling was originally developed to assess issues related to global change, such as climate change, and lends well to analyzing water supply development within the Earth system. System dynamics simulation provides a practical approach to implementing integrated assessment models.

## Model description

The highly endogenous structure and coupling of sub-systems in the ANEMI3 model are part of its novelty in the realm of integrated assessment modelling. Because of this, feedback processes are responsible for the behaviour that is exhibited in model runs. The ANEMI3 model builds from its previous version, ANEMI2 [12]. The significant structural modifications of the model include: (i) implementation of the energy-economy sector based on the principles of system dynamics simulation; (ii) incorporation of water supply as an additional sector in the global economy that parallels the production of energy; (iii) inclusion of climate change effects on land yield and potentially arable land for food production, and (iv) addition of nitrogen and phosphorus based nutrient cycles as indicators of global water quality, which affect the development of surface water supplies. Additional modifications to the food production sector are included, and a persistent pollution sector is incorporated that acts to limit population growth and food production. The model sectors that comprise the ANEMI3 model are climate, carbon, nutrient, and hydrologic cycles, population dynamics, land use, food production, sea level rise, energy production, global economy, persistent pollution, water demand, and water supply development. Feedback loops between sectors, or intersectoral feedback loops are responsible for global change in this Earth system. This work focuses on representing global scale feedbacks that are driving global change and assessing their importance and influence within the Earth system. However, there are processes occurring at finer spatial scales that have global impacts which cannot be represented by ANEMI3. In the following sections of the paper the model sectors and their structure are introduced, along with the driving feedbacks created by their connections. The mathematical details are available in the report by [17]. The report presents the ANEMI3 model structure in detail and provides discussion of parameter estimation and model validation.

**Fig 1:**
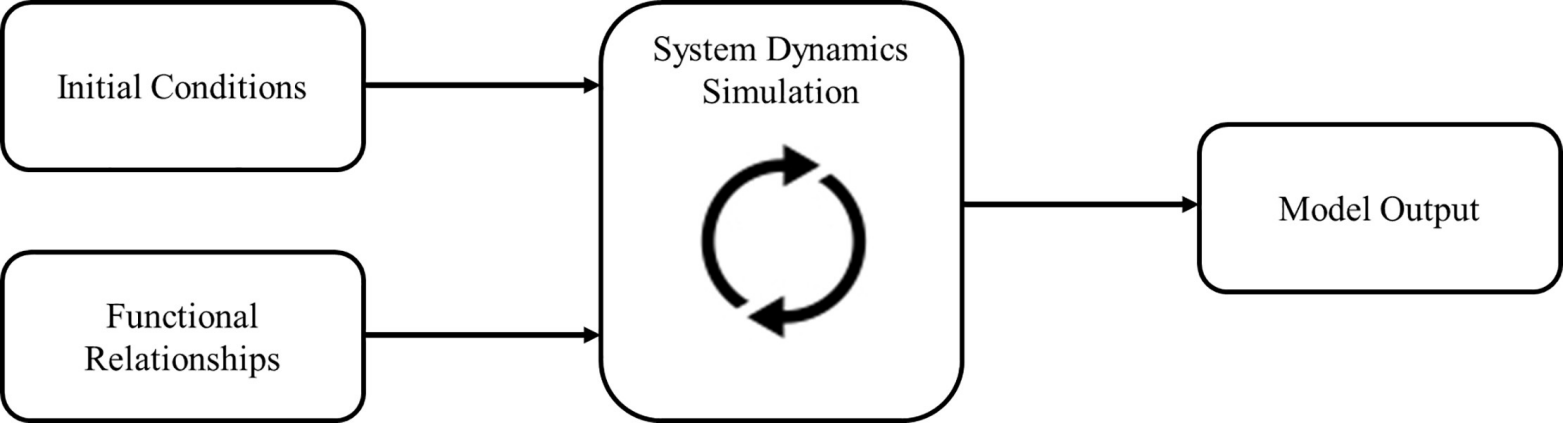
High level flowchart for simulation of ANEMI3 using Vensim system dynamics simulation software.

The model was developed using Vensim version 6.3 [18]. The model time horizon is 100 years with a time step of 1/128^th^ of one year, with annual values provided as output. The Vensim software was chosen for development as it is designed for the construction of system dynamics simulation models. The software allows for the structure of the simulation models to be viewed graphically in the form of stock and flow diagrams and allows for feedback loops to be easily identified in large systems of many interconnected variables. At a high level, simulation of the model in Vensim follows the flowchart in Fig 1. Initial conditions for each stock or state variable are set prior to simulation of the model, along with any functional relationships described using “look-up” tables which use linear interpolation to relate two sets of data points. The system of equations built with the Vensim software are then integrated using a solver of choice. Model outputs are saved and stored following the simulation.

The entire model code is archived using Zenodo (https://doi.org/10.5281/zenodo.4483736) [19]. Details on running the model, modifying inputs, and viewing the outputs in graphical or table formats are provided in the repository. The web version of the program, GCE (Global Change Explorer), allows for the model to be simulated through a series of interactive, user-defined scenarios and is available at https://globalchange-uwo.ca/ (last accessed January 24, 2021). It can be also found on the System Dynamics Society’s case study repository, https://systemdynamics.org/list-of-all-cases/ (last accessed January 24, 2021).

### Intersectoral feedbacks

The model sectors that compose ANEMI3 are selected to represent the dynamics of global change at the global scale, emphasizing the development of water supplies. Here, water supply is defined as the rate of water available to satisfy water demands. This water is extracted from different types of available water resources including surface water, groundwater, desalination and water reuse. It is assumed that water supply is constrained by infrastructure needed to process water from a given source (extraction, distribution, and treatment). For example, although the ocean provides a vast amount of available water resources, the rate at which water can be supplied from this source is constrained by desalination infrastructure. Water stress is commonly represented as the ratio of water withdrawals to available water resources. In the ANEMI model, the ratio of water withdrawals (or demand in this case) to the available water supply is used to capture the development of water supplies and its effect on water stress.

**Fig 2:**
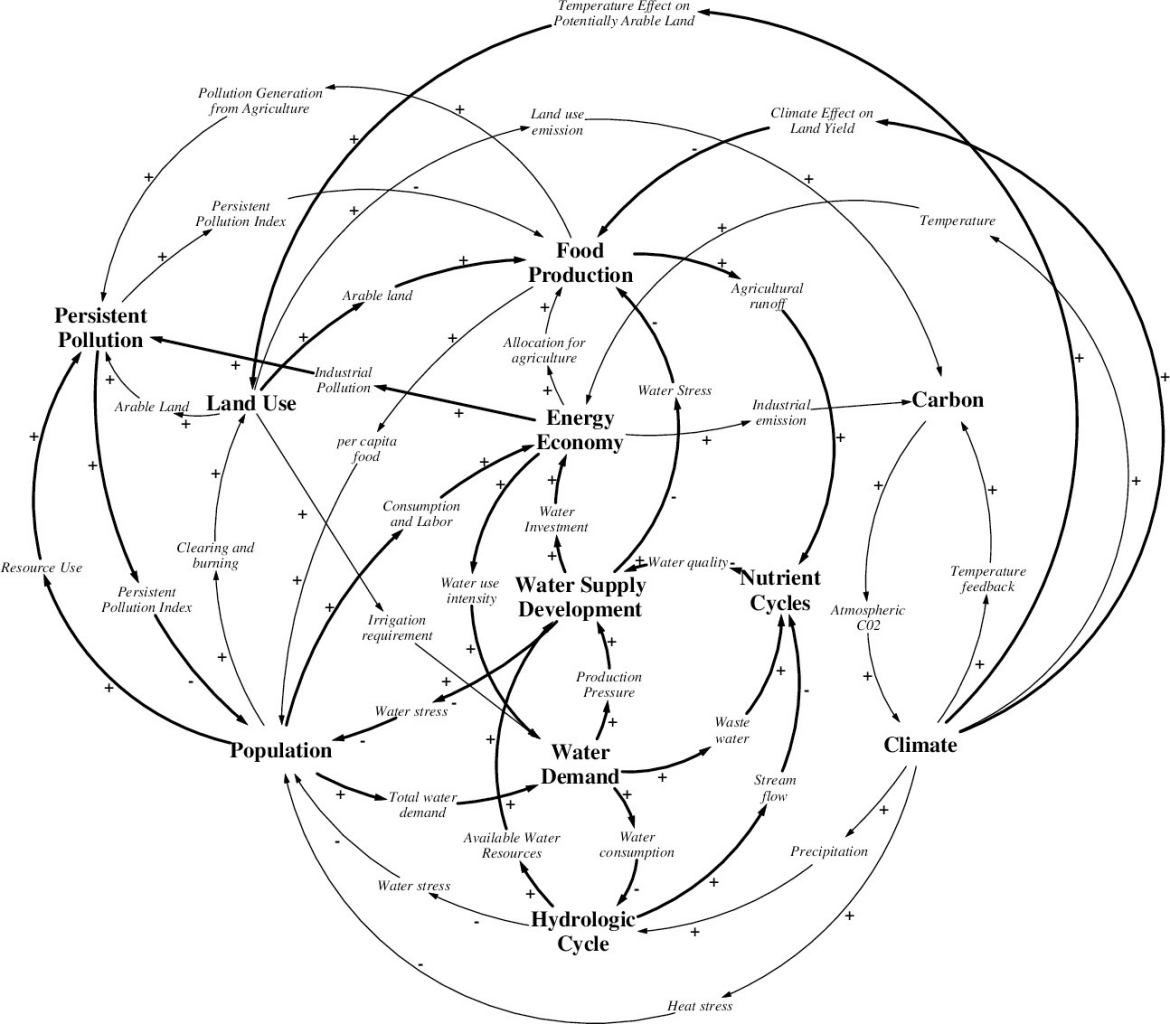
High-level feedback structure of the ANEMI3 model illustrated as a causal loop diagram. Bolded arrows denote new Earth system feedbacks added in ANEMI3.

Creating a causal loop diagram from connections between model sectors allows us to view the feedbacks that are created by combining model sectors in this way (Fig 2). Intersectoral feedbacks in the ANEMI3 model allow for the representation of various aspects of global change. In this diagram alone there is a total of 89 possible intersectoral feedback loops. The size of the feedback loops range from 2 to 9 sectors included out of the 11 that are shown.

By organizing the components of the Earth system in this way, feedback processes that drive global change can be represented. An example is that of a growing global economy, which drives energy production and industrial growth, thereby resulting in more greenhouse gas emissions and climate change. This in turn results in negative feedbacks on economic growth through climate damages, which can represent economic damages as a result of land and structures lost to coastal flooding, for example. This feedback loop is present in other feedback-based integrated assessment models. With the modified feedback structure of the ANEMI3 model in this work, global scale feedbacks are created in addition to those present in the previous iteration. The main changes to the model structure from ANEMI2 are listed below and illustrated in Fig 2 (bolded arrows):

- Water supply development allows for increased water use intensity and water consumption. This in turn reduces the amount of available water resources, which has a limiting effect on water supply development.
- Increased water supply development results in a decrease in water stress, allowing for more population growth and water demand, thereby increasing production pressure on water supply.
- Investment in water supply capital stocks increases the global aggregate capital stock, thereby increasing water usage intensity and water demand, creating more pressure for water supply development.
- The development of water supplies alleviates water stress on food production, allowing for more food to be produced and thereby increasing agricultural runoff to the nutrient cycles. This, in turn, negatively impacts water supply through reduced water quality and increased surface water supply costs.
- Persistent pollution adds additional negative feedbacks to population growth by acting as a multiplier of life expectancy. With increased population, the total use of natural resources and pollution generation increase. This increases persistent pollution levels over time, resulting in reduced population growth.
- Increased population has a positive effect on the global economy by boosting the labor force, resulting in more industrial pollution. This in turn has a limiting effect on population growth through the life expectancy multiplier from persistent pollution.
- Increased population also provides more labour input which supports economic development. Overall, greater economic development generally results in reduced water withdrawal intensities in the domestic and industrial sectors through technological improvements (i.e., through the application of water saving appliances and more water efficient industrial processes), resulting in less water consumption and more available water resources. This enables more water supply development thereby limiting water stress and supporting further population growth.
- Rising temperature can potentially reduce agricultural land yields, thereby limiting food production. In addition, rising temperatures may also result in greater arable land in northern regions, increasing the potential level of food production. Both effects occur simultaneously, and the net effect is dependent on the amount of global temperature increase and other factors affecting land yield and arable land.

Viewing the number of intersectoral connections to and from each sector provides an indication of their degree of coupling within the ANEMI3 model. If a sector has no outgoing connections and only incoming connections, there would be no potential for feedbacks, and it would mainly be for assessment purposes. A sector with a high number of incoming and outgoing connections is likely to have more intersectoral feedbacks. Finally, if a sector has all outgoing connections and no incoming connections then it can be considered as exogenous input to the model. The sectors with the highest number of combined incoming and outgoing connections are population, economy, climate, and food production indicating that they have a high degree of connectivity to other sectors in the model and potentially more feedbacks. An overview of all the model sectors is provided in the next section, along with details on the structural modifications made in ANEMI3.

### Overview of model sectors

#### Climate

**Fig 3:**
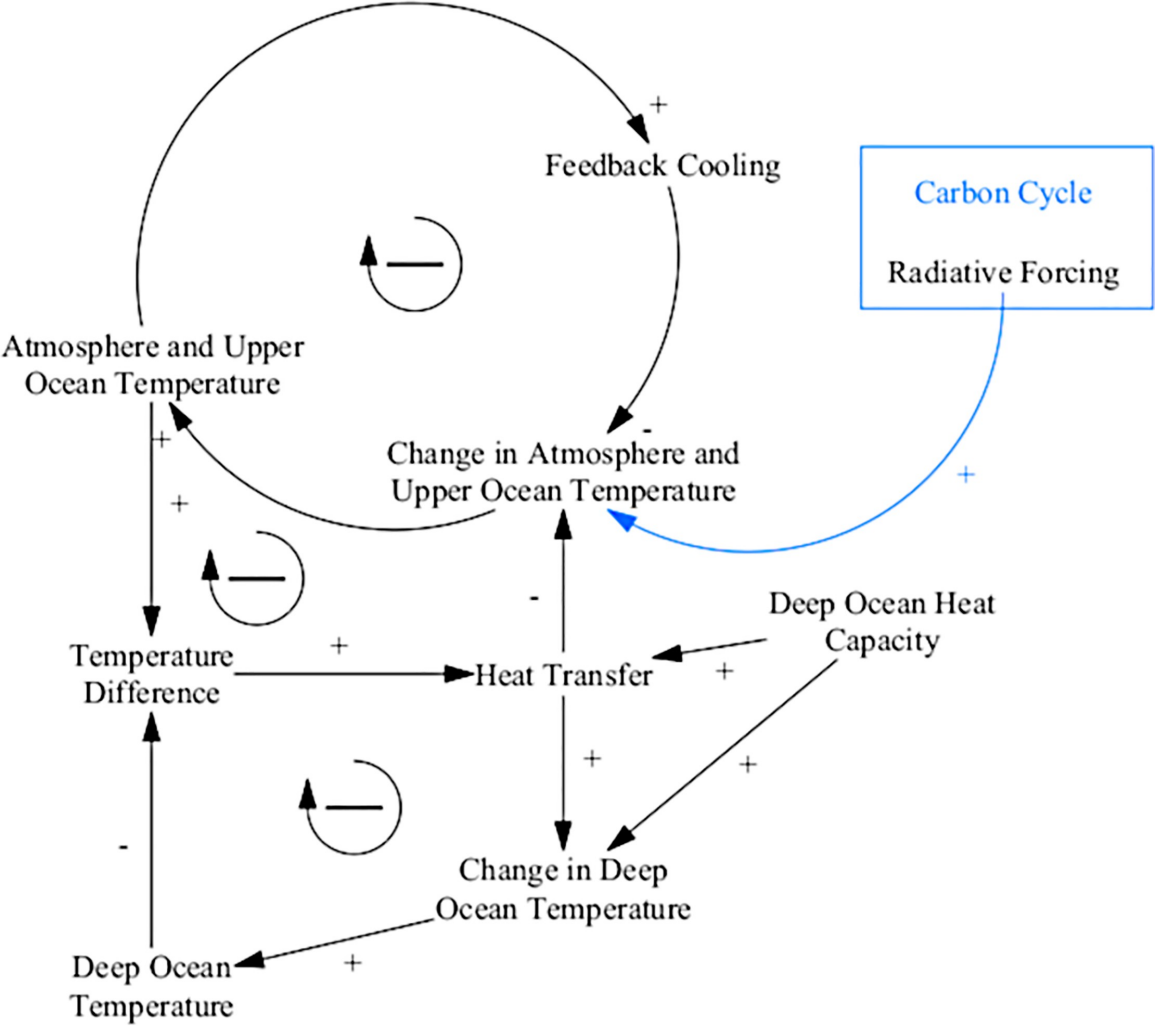
Causal loop diagram of the ANEMI3 climate sector. Coloured arrows and boxes represent connections to other model sectors.

The climate sector of ANEMI3 is based on the DICE model [20] and is not significantly modified in comparison to previous versions. In this sector, the dynamics of heat exchange between the deep ocean and the combined upper ocean and atmospheric layers are modelled, along with a cooling effect that acts to limit the rate of temperature increase. Identifying the feedbacks that drive this simple climate system allow us to speculate on how the system will function over time. The climate sub-system is driven by two feedback loops (Fig 3). The first being a feedback cooling effect, while the second represents the diffusion of heat in the atmospheric stock to the ocean stock. These negative feedbacks act to dampen the response of the system to radiative forcing which comes from increased greenhouse gas concentrations in the carbon cycle and greenhouse gas sub-systems. Based on this simplified climate system structure, one might expect it to predict global temperature values on the lower end of the spectrum. This is because positive feedbacks related to climate change such as methane release from tundra regions and change in albedo as global ice cover melts (currently limitations of the carbon cycle sector) are omitted and have the potential to accelerate increases in global temperatures.

#### Carbon cycle

The carbon cycle in the ANEMI3 model is used to represent the flow of carbon through the Earth system from the atmosphere to land and oceans [21] and it maintains a similar structure to previous versions of the model. By incorporating the entire carbon cycle, atmospheric concentration can more realistically be simulated to drive changes in climate through the greenhouse effect. Feedbacks between the carbon cycle and climate system can also be represented through increased solubility of carbon dioxide in the ocean and fertilization effects of plant material with increased global temperatures. Finally, by modelling the cycle of carbon, connections can be made with the land-use sector by separating the land stock of carbon into different biome types. This allows for changes in land use such as the burning and clearing of grasslands and forests, to contribute CO_2_ emissions to the atmosphere.

**Fig 4:**
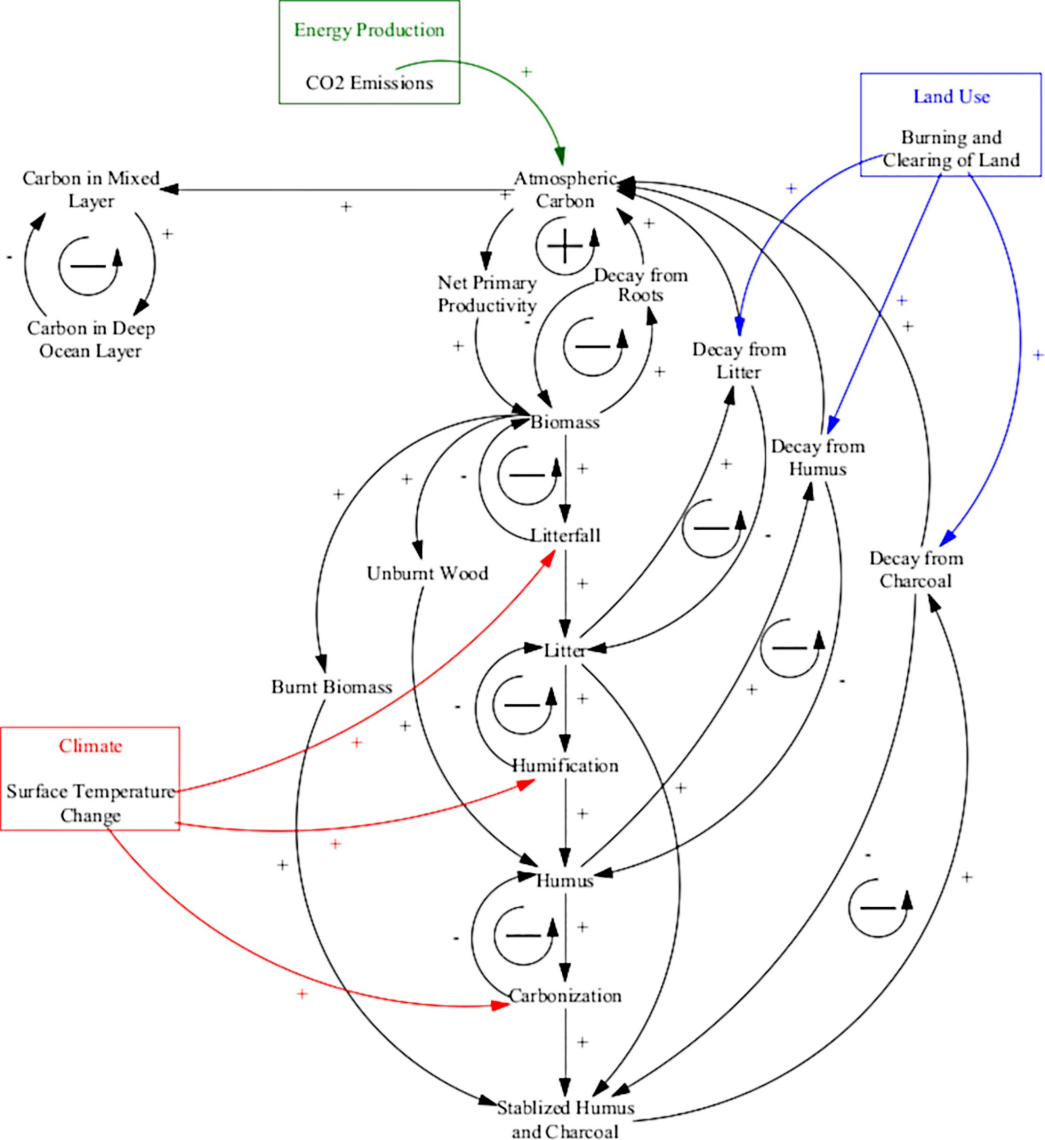
Causal loop diagram of carbon cycle sector in ANEMI3. Coloured arrows and boxes represent connections to other model sectors.

The causal loop diagram of the carbon cycle sector is given in Fig 4. The chain of negative feedback loops passing through each of the terrestrial carbon stocks from the atmosphere and back again act as a positive feedback loop in the carbon cycle. This is because more atmospheric carbon results in higher uptake of carbon in the biomass, which results in the greater transfer of carbon through the chain (litter, humus, stabilized humus and charcoal) thereby resulting in more decay and transfer of carbon back to the atmosphere. Although these are positive feedback loops, carbon in this cycle is conserved, but the release or storage of carbon in the terrestrial stocks will be dependent on the balance between uptake and decay. The last feedback loop in the diagram is a negative feedback loop that represents the diffusion of carbon dioxide between the two ocean layers. Methane release from tundra regions and change in albedo as global ice cover melts are not incorporated in the current version of the model and can be considered as limitations to be addressed in future work.

#### Population

**Fig 5:**
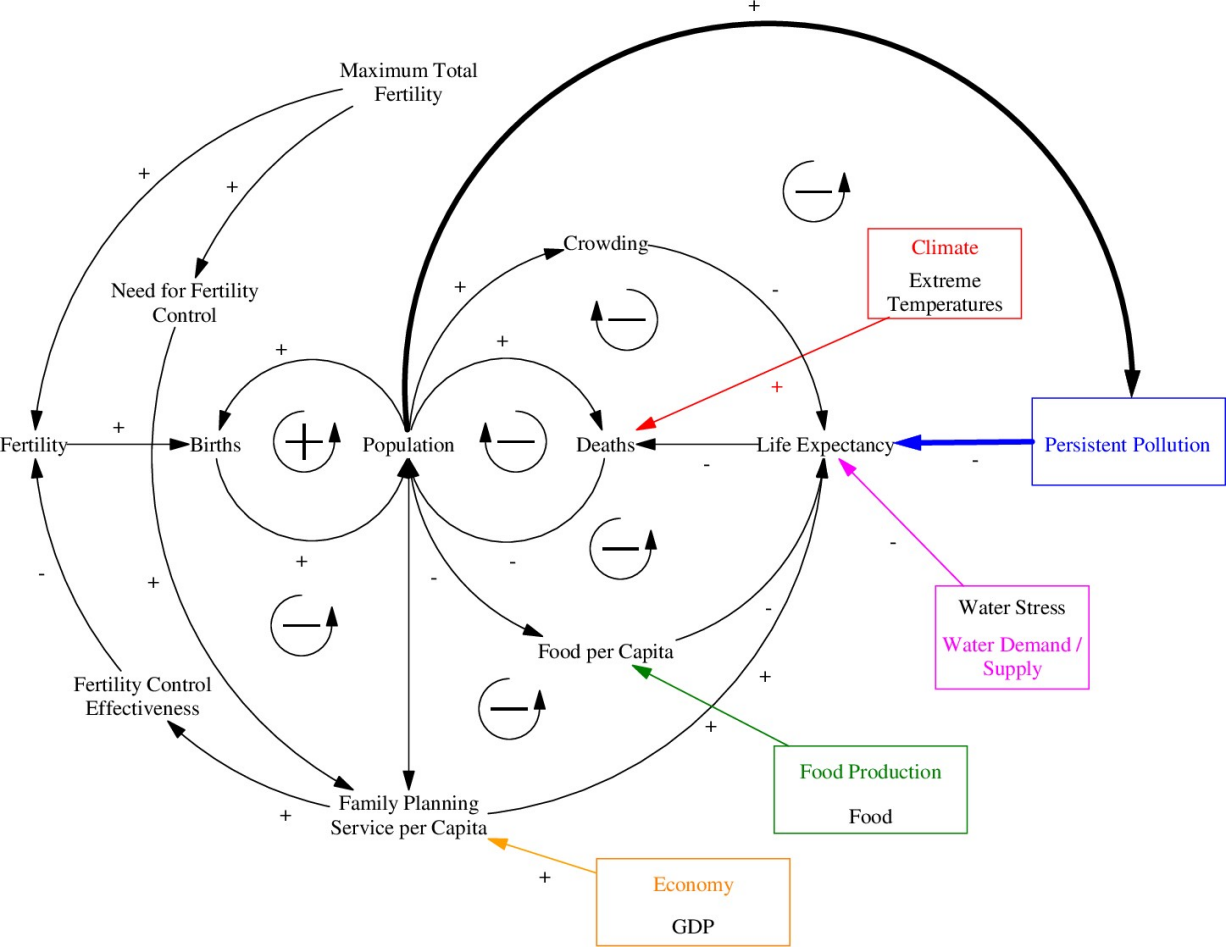
Causal loop diagram of the ANEMI3 population sector. Coloured arrows and boxes represent connections to other model sectors. Bolded arrows denote causal relationships implemented in ANEMI3.

The causal loop diagram in Fig 5 illustrates the feedbacks associated with the population sector, which maintains the same form as previous versions of the model. One positive feedback loop drives the system and is responsible for the exponential growth of the human population. A higher population results in a higher growth rate through more births and therefore a higher population. The rest of the population sector details a series of negative feedbacks that limit population growth. The negative feedbacks include the effects of crowding, water stress, extreme temperatures, food production, persistent pollution, and wealth represented as global GDP. All of which are always active but to different degrees and affect either the life expectancy and thus mortality rates of the population, or fertility thereby reducing birth rates. Each of these effects act as multipliers and are related through look-up tables that could be associated with a significant amount of uncertainty.

The population sector of ANEMI3 uses separate stocks to split the population into different demographics of ages 0 to 14, 15 to 44, 45 to 65, and 65+. This was done to capture the effects of delays in demographic responses to external conditions, thereby affecting birth and death rates. It allows for the growth of the total population to retain some inertia as external conditions change which more closely captures the dynamics of population growth in the real world. This structure also allows for the population of different age groups to be used in other model areas. For example, the 15 to 44 and 45 to 65 population groups are combined and used as the labour force in the energy-water economy sector. Another reason as to why these groups were used is so that age group specific factors that influence mortality can be applied. Climate change is included as an influence on mortality rate using a temperature multiplier that acts to influence deaths due to the presence of more frequent heat waves causing heat stress. Factors influencing fertility and birth rates are also included through socioeconomic drivers.

An increasingly important dynamic that is currently not included in the ANEMI3 model is the migration of the human population driven by climate change. It has been estimated that the number of climate migrants could reach 200 million by the year 2050 as a result of shoreline erosion, coastal flooding, and agricultural displacement [22]. Climate migration on such a scale could have far-reaching effects on all aspects of the Earth system.

#### Land use

**Fig 6:**
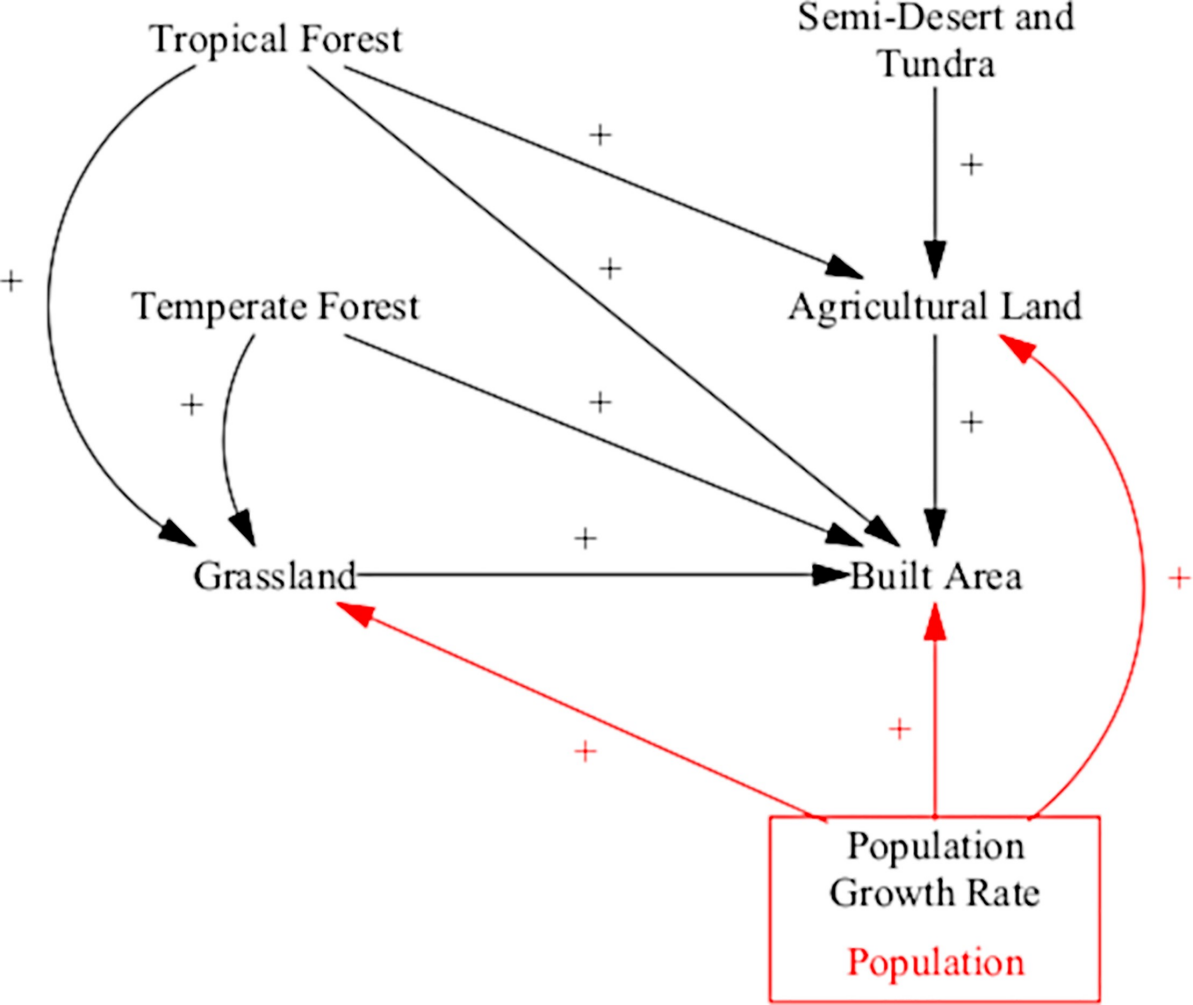
Causal diagram of the ANEMI3 land-use sector. Coloured arrows and boxes represent connections to other model sectors.

The land-use sector is used to describe the global distribution of land use and cover over time. This is done by modelling the rates at which one land use or cover type is changing into another. Six land use and cover classes or biome types are used: tropical forest, temperate forest, grassland, agricultural land, semi-desert and tundra, and urban area. Accounting for changes in land use and cover is an important component in ANEMI3. It determines the conversion of land for agricultural purposes and thus the production of food to support growing populations. The main change from the previous version of the model that is incorporated in the ANEMI3 includes accounting for a release of CO_2_ as one land type converts to another. For example, as forests are converted to agricultural land there is a release of CO_2_ associated with the loss of vegetation, which makes the effect of land cover change an important source of CO_2_ emissions in the model contributing to the greenhouse effect. The causal structure of the land use sector is shown in Fig 6.

#### Food production

**Fig 7:**
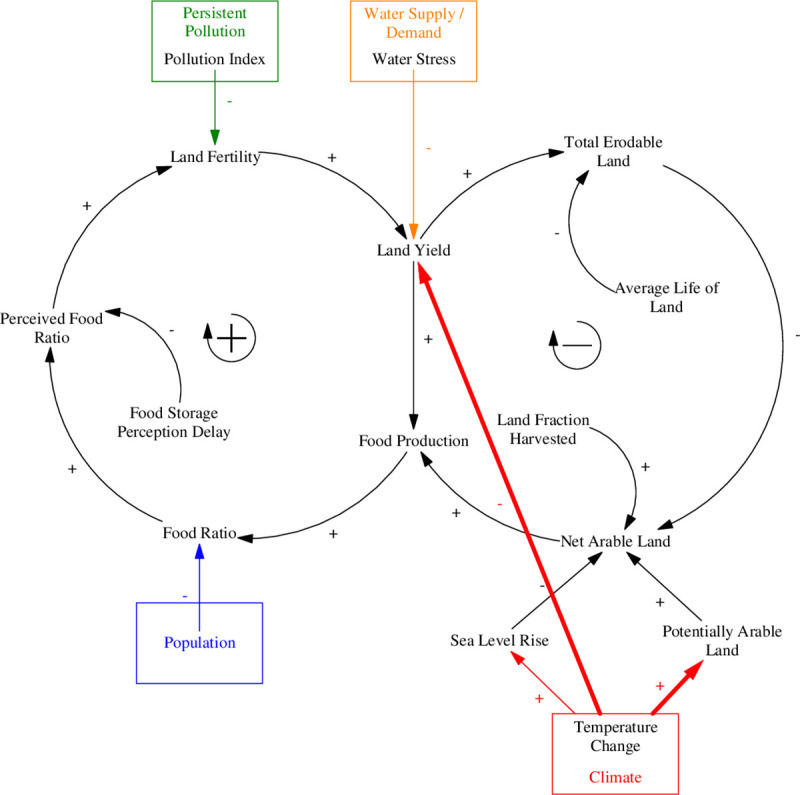
Causal loop diagram of the ANEMI3 food production sector. Colored arrows and boxes represent connections to other model sectors. Bolded arrows denote causal relationships implemented in ANEMI3.

The food production sector in ANEMI3, models global food production, which is ultimately used to determine the level of food per capita as an indicator for limitations to population growth. The production of food is affected by several factors including land fertility, arable land, water, and nutrients. The food production sector is based on that of the WORLD3 model [23]. The most significant contribution to the ANEMI3 food production sector is in the explicit consideration of climate change impact. Climate change impacts on food production in ANEMI2 were limited to reductions in arable land due to sea level rise. In ANEMI3, additional climate change effects are incorporated through impacts of rising temperatures on land yield due to heat stress [24], and potential increase of arable land due to the northward expansion of viable agricultural areas [25]. The feedback structure of the ANEMI3 food production sector is shown by the causal loop diagram in Fig 7. Two main feedback loops drive food production. The positive loop represents the effect of increased food production driving more reinvestment in increasing land fertility and thus food production again. The negative loop represents decreasing land yield due to food production, leading to more land erosion and then less arable land available for food production.

Food production can be altered in two ways through this representation. Either the amount of arable land can be increased by cultivating more land for agriculture, or the yield of that land can be increased through the application of modern agricultural inputs. In ANEMI3, climate change through increased temperatures can affect the level of potentially arable land, as changes in the number of growing days available in a given region can allow for agricultural activities to become feasible in areas where they were not. Two main factors limit the food production in the model. The first is that food production is reduced by land erosion, which limits the ability to produce food from the stock of arable land. The second is reduced land fertility, which arises from water and heat stress as well as pollution.

#### Hydrologic cycle

**Fig 8:**
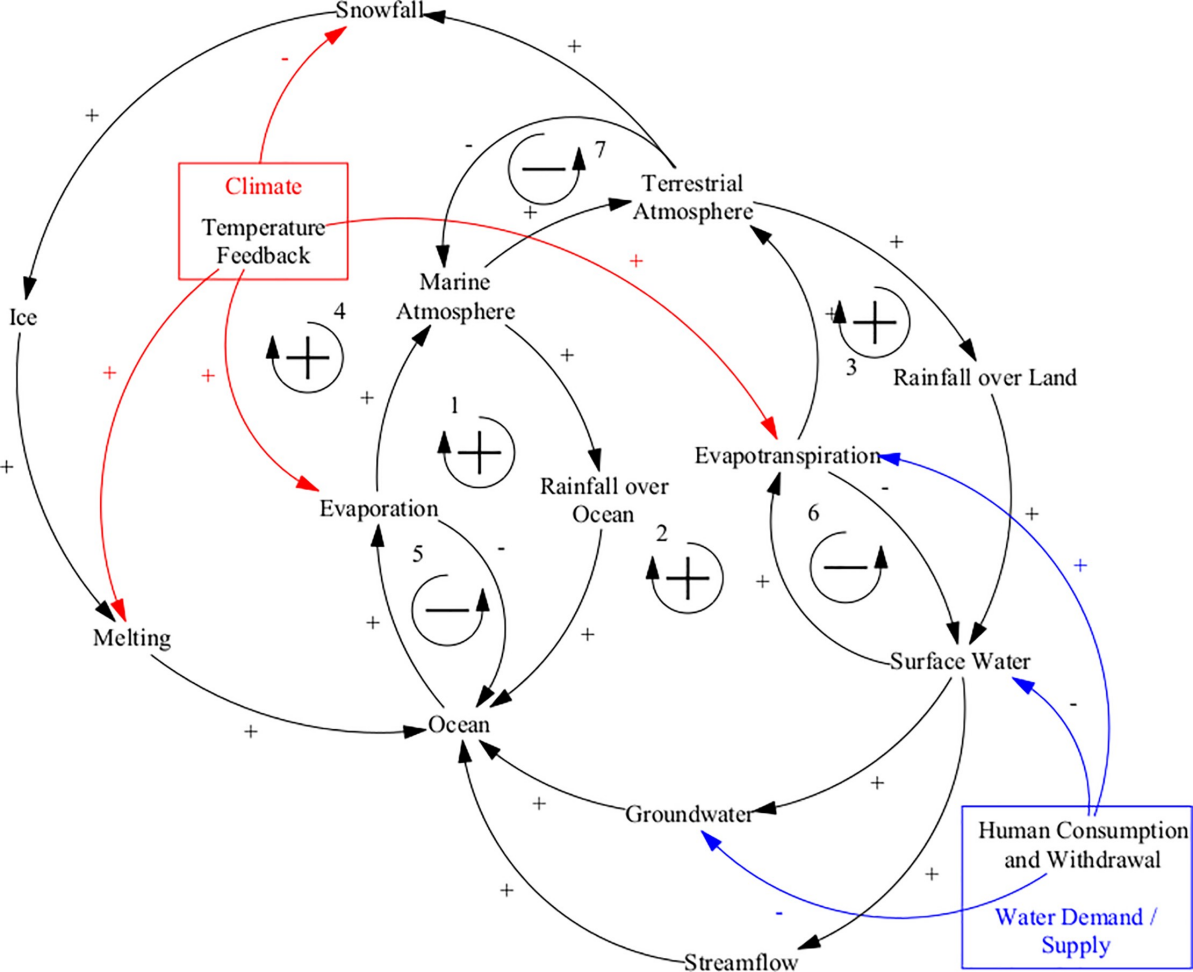
Causal loop diagram of the ANEMI3 hydrologic cycle sector. Colored arrows and boxes represent connections to other model sectors.

The hydrologic cycle describes the flow of water from oceans to atmosphere, onto the land surface and through the groundwater back to the ocean again as a continuous cycle. The ANEMI3 structure of the hydrologic cycle sector is the same as in ANEMI2. Each point in the hydrologic cycle can be considered as a kind of reservoir from which water flows to and from. The causal loop diagram in Fig 8 illustrates the feedback loops at work that drive the hydrologic cycle. Feedback loops number 1–4 in Fig 8 illustrate the movement of water from the atmosphere (terrestrial or marine) to the surface (ocean or land) as rainfall or snowfall and then back to the atmosphere (through evaporation and evapotranspiration). These are positive feedback loops because more water in oceans and surface waters results in a larger surface area increasing evaporation which drives up atmospheric moisture leading to more rainfall then more water in oceans and surface waters once again. The positive loops are balanced by negative loops 5 and 6 which regulate increases in land and ocean water volumes by increased evaporation. Loop number 7 illustrates the balance between advection of atmospheric water over oceans and land surfaces as this process depends upon the difference in water content between them.

#### Energy economy

The energy-economy sector used in ANEMI2 was based on the traditional Solow neoclassical growth model where economic output is represented as a function of capital and labor in the form of a Cobb-Douglas production function [26]. The growth of capital is dependent on investment, which is determined by a Solow rule where a fraction of output is reinvested in new capital every time period, while population growth increases the labor force, thereby boosting output and the capital stocks over time. This reinforcing behaviour on the output is combined with a partial equilibrium model where the global economy consists of a representative household and a representative firm. The representative household encapsulates the World’s population, whose preferences are captured by a utility function based on consumption. The household generates income by renting capital and selling energy services to the firm and earning income from the labour force. This income provides a budget constraint to the household for which it maximizes its utility function. On the other hand, the firm seeks to minimize the total cost of producing energy amongst different sources. As these two dynamics unfold, prices for energy production move to clear the market and achieve equilibrium between energy supply and demand for each time step. The structure of this model allows for the examination of long-run economic growth of aggregate capital stock as well as the production paths for fossil fuels and renewable energies.

The new energy-economy sector in the ANEMI3 model is based on the Feedback-Rich Energy Economy model (FREE), which brings together feedback-based models of energy production and economic development [27–29]. The ANEMI3 model structure of the energy-economy sector is using a completely different approach from the previous versions of the model. The implementation of the energy-economy system in ANEMI3 is based on the principles of system dynamics simulation. Many of the dynamics related to economic growth and resource depletion from the previous approach remain, but some key structural differences exist. The first is that the macroeconomic assumption of market equilibrium used previously is no longer present, as the model being used here is a disequilibrium model. Instead of energy prices being set to equate supply and demand at every time step, there are negative feedbacks that constantly drive supply to meet the demand as they change over time.

**Fig 9:**
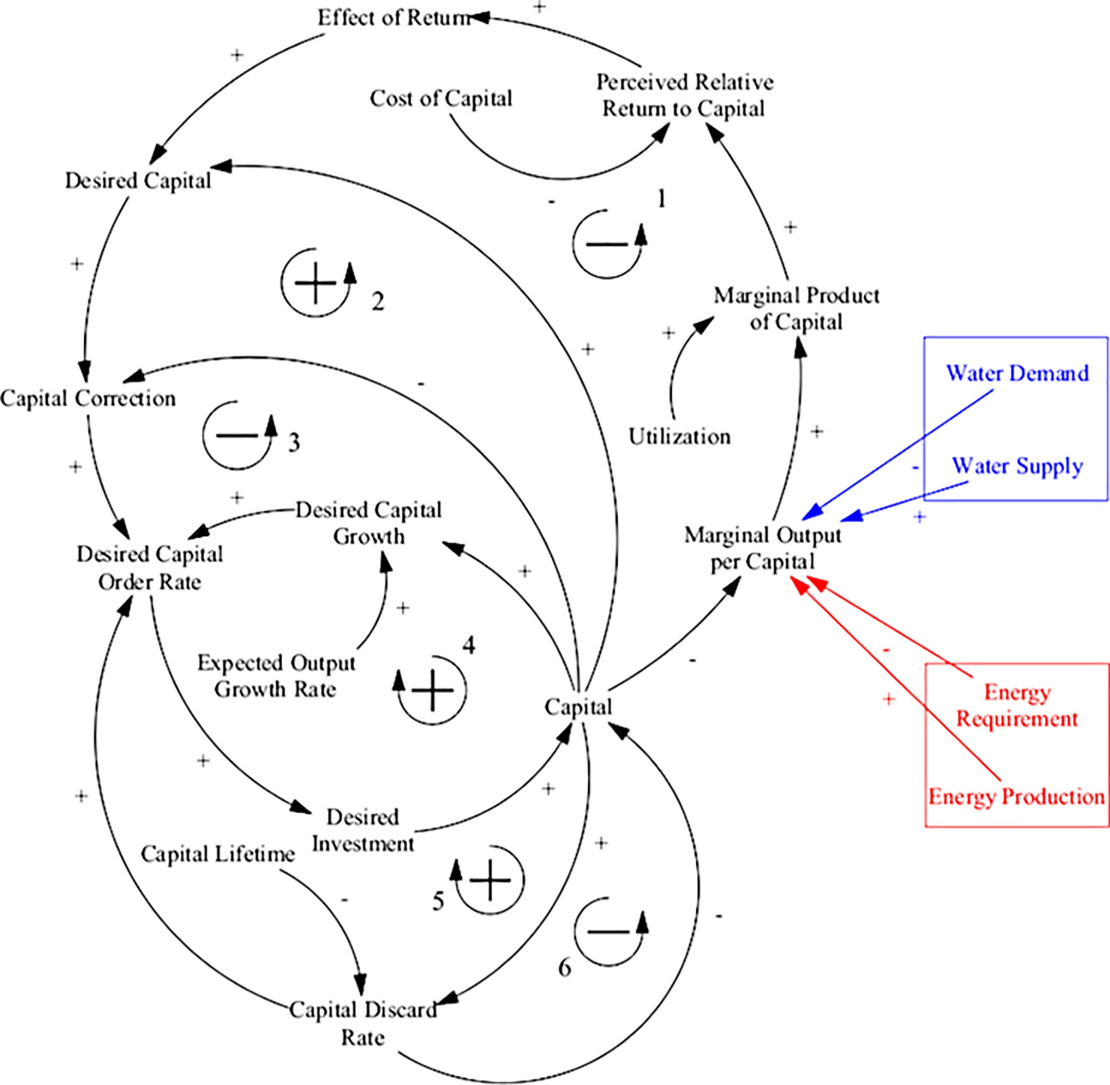
Causal loop diagram for good production and capital sub-system of the energy-economy sector. Coloured arrows and boxes represent connections to other model sectors. The entirety of this model sector is a new addition in ANEMI3 used to replace the previous energy-economy system of ANEMI2.

The dynamics of the aggregated capital stock of the global economy is shown in Fig 9, consisting of five main feedback loops. The first and second loop depict the adjustment of the desired capital stock in response to the relative cost and marginal product of capital. The gap between the desired and actual capital stock is corrected in the third loop. The fourth loop illustrates the incorporation of expected output growth rate on investment, and the fifth loop factors capital depreciation into investment in additional capital.

Energy is produced to meet the demands for the production of goods and services (i.e., economic output). The production of energy is disaggregated into four types: coal, oil and gas, hydro and nuclear power, and renewables. Hydro and nuclear energy sources are combined into a single energy source because they have similar carriers (i.e., generation of electricity to a grid) and long-term characteristics, including diminishing returns to expansion as the best sites are used first and are subject to political and environmental constraints [27].

**Fig 10:**
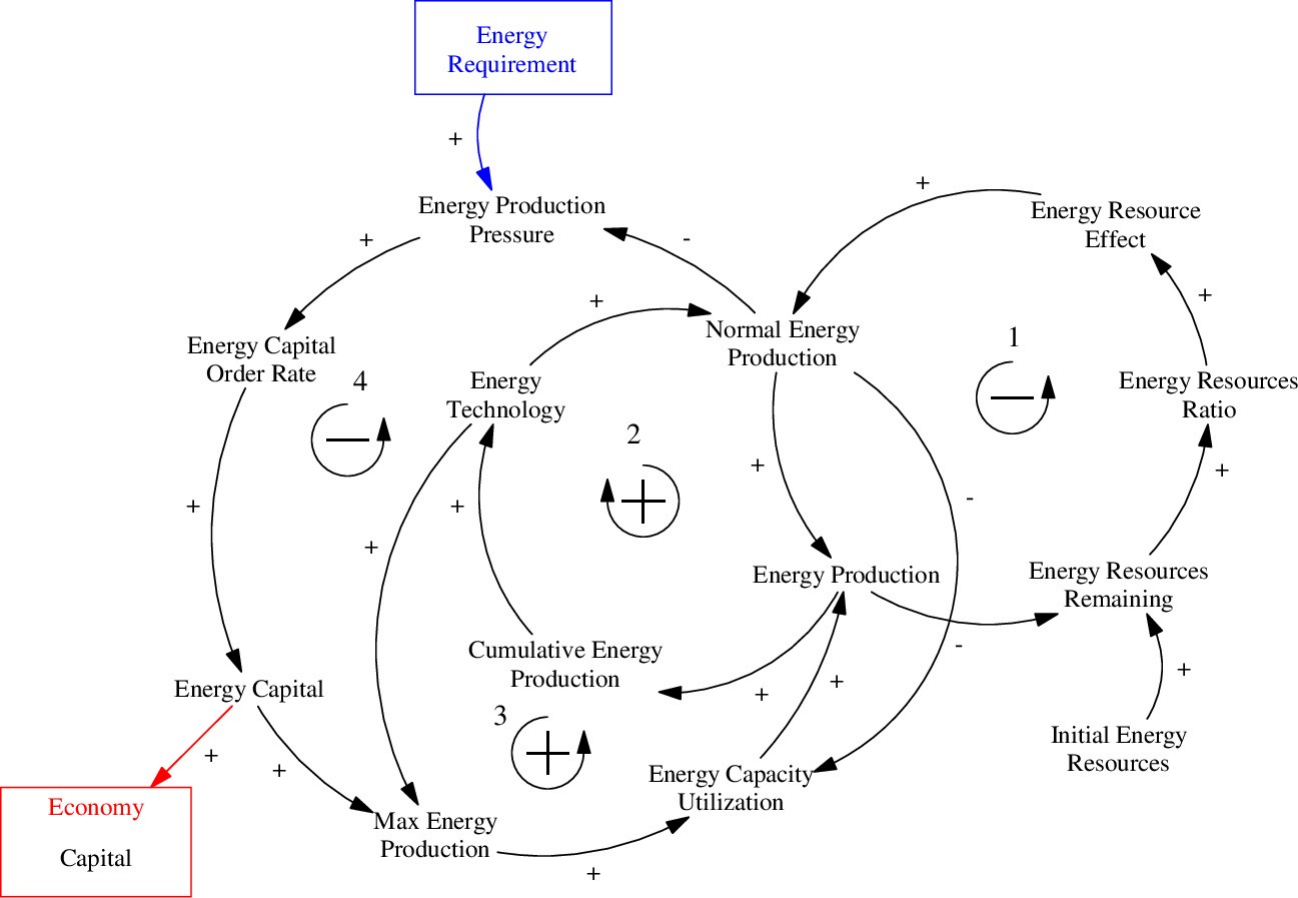
Causal loop diagram for the energy production sub-system of the ANEMI3 energy-economy sector. Coloured arrows and boxes represent connections to other model sectors.

The capacity of energy production is set by the amount of capital stock that has been accumulated into each energy source and is influenced by production pressures and profit incentives. The rate of variable inputs determines the utilization of production capacity. Limitations on energy production are in the form of depletion and saturation for non-renewable and renewable energy sources. Depletion refers to the use of limited resource stocks (i.e., fossil fuels), thereby increasing the effort and cost required to extract the resources. Saturation in this context refers to diminishing returns to energy production effort. For example, the ideal sites are taken first to implement wind and solar farms or dams for hydropower generation, thereby making it more difficult and/or expensive to implement additional sites. These concepts are illustrated in the causal loop diagram in Fig 10.

Feedback loop number 1 illustrates the effect of resource depletion on energy production. As more energy is produced, energy resources begin to deplete. This affects the ratio of energy resources remaining, which reduces energy production, creating a negative feedback loop. The second loop is a positive loop, which illustrates the increasing efficiency of energy production through technological improvements over time, driven by cumulative energy production. The third loop represents the perpetual production of energy to meet demand. As energy is produced, resources begin to deplete, causing a reduction in production through the resource depletion effect. This in turn causes production pressure to meet demand, resulting in further investment in energy capital stocks, thereby increasing production again. The fourth loop is a negative feedback loop, which limits the increase in energy production as technological improvements are made, thereby boosting energy production and reducing production pressure.

The capital stocks for the different energy sources are structured in a similar way to that of the goods production capital stock. The main difference is that there is a stock that represents energy capital under construction which after a time delay becomes new energy capital.

**Fig 11:**
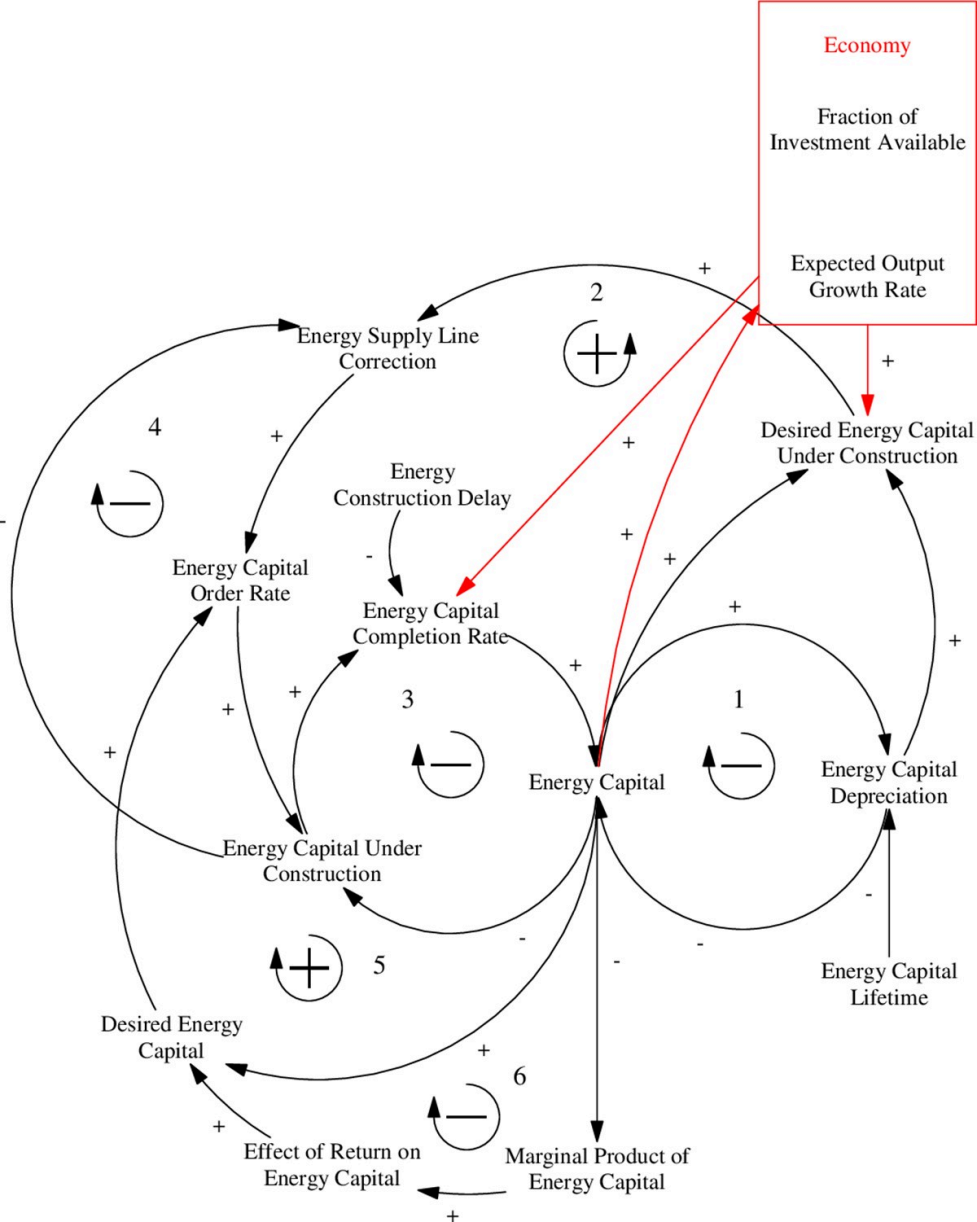
Causal loop diagram for the energy capital sub-sector of the energy-economy sector.

There are six feedback loops in total in the energy capital sector (Fig 11). The first loop is a negative feedback loop that drives the process of energy capital depreciation, which slowly depletes the energy capital stock. The second loop, being a positive feedback loop compensates for depreciation by factoring it into the desired energy capital order thus boosting the energy capital order rate and energy capital. The third loop moves energy capital from the construction phase to the completion phase. The fourth loop reduces energy orders by taking into consideration capital that is currently under construction when determining the desired energy capital order rate. The fifth loop is a positive feedback loop that increases capital investment based on perceived returns. The sixth loop reduces the effect of perceived returns, thereby limiting the positive effect of the fifth. This is because more energy capital results in reduced marginal product of capital, thereby reducing the return on energy capital investment. These feedback loops, in combination, drive the energy production of the ANEMI3 model.

One of the unique features of the FREE model in contrast to other climate-energy-economy models of its kind is the embodiment of energy requirements, or demand, in the capital stock [27]. This means that when capital is constructed, it has a fixed energy intensity. In the real world, this equates to energy consumption being dependent on products that persist with time. For example, once an electric stove is manufactured its energy efficiency cannot be changed. This contrasts with other models like DICE, which assume that appliances like an electric stove could be converted to one that uses natural gas [20]. In the FREE model, transitioning between energy sources requires the gradual substitution of energy capital due to price changes even if the current allocation of capital is suboptimal.

#### Water demand and supply

**Fig 12:**
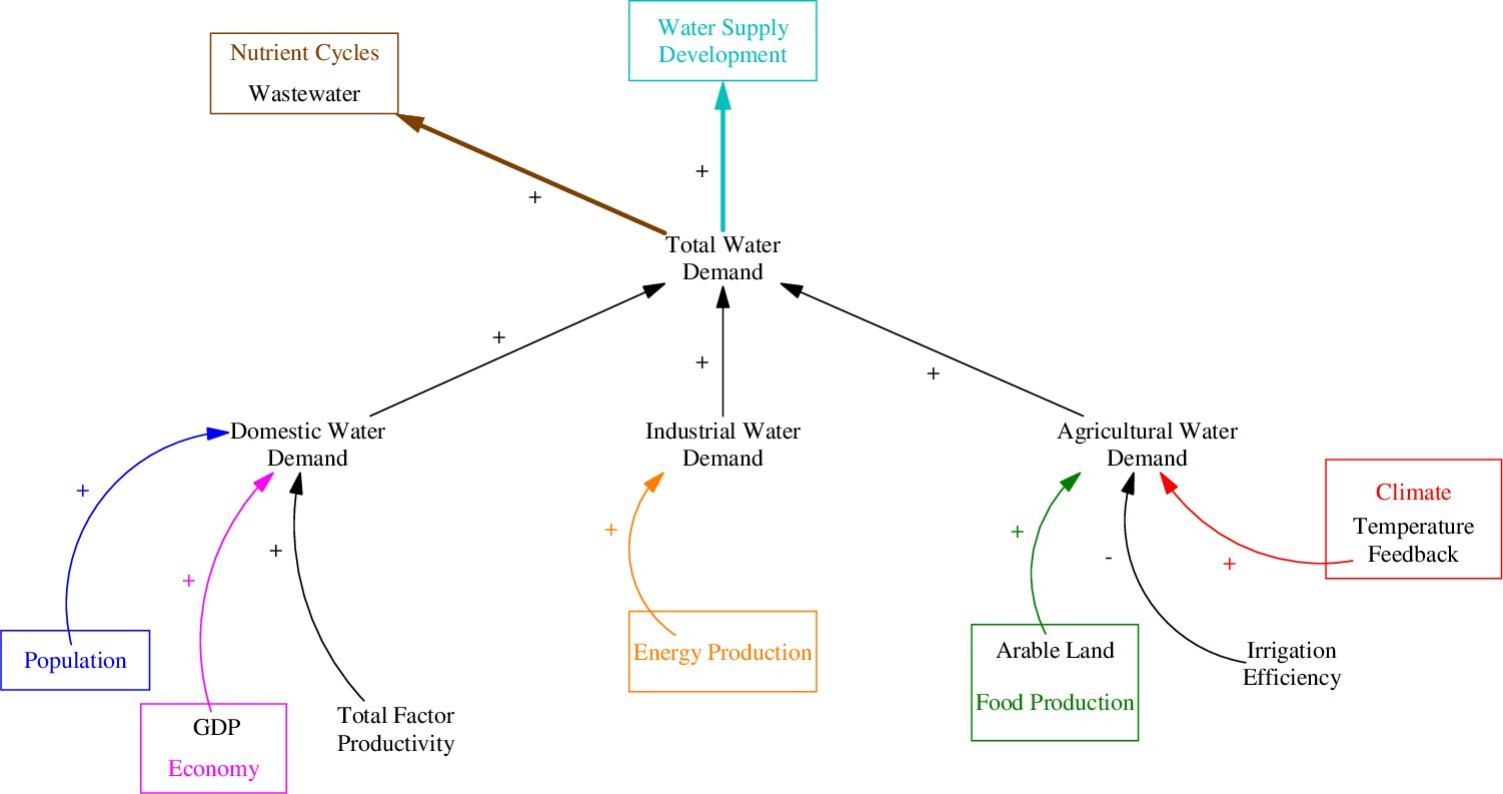
Causal diagram of the ANEMI3 water demand sector. Colored arrows and boxes represent connections to other model sectors. Bolded arrows denote causal relationships implemented in ANEMI3.

The water demand sector in the ANEMI3 follows previous versions of the model and is based on the desired water withdrawals of agricultural, domestic, and industrial water users. Domestic water withdrawals depend on structural water intensities that relate economic factors such as GDP to withdrawal rates per person [30]. This concept has been confirmed in historical data for countries of different sizes and levels of GDP [31]. The relationships presented indicate that there are established trends in water usage as countries become developed using economic development indicators such as GDP per capita. Domestic water use in terms of water volume per capita tends to increase as more water is needed for improved sanitation and use of more water-using appliances such as dishwashers and washing machines. This trend stabilizes in the developed countries. The causal diagram in Fig 12 shows the water demand sector including domestic, industrial, and agricultural water users. Although there are no feedback loops within the water demand sector itself, there are many intersectoral connections and feedbacks associated with water demand.

In ANEMI2, water supply was treated the same way as available water resources. Conventional water resources such as surface and groundwater resources were consumed in response to demand, and alternative water resources such as wastewater reuse and desalination increased exogenously in response to water stress. In ANEMI3, water supply infrastructure is developed over time through economic investment in surface water and groundwater resources. This distinction allows for alternative water resources to be developed not in response to water stress, but to increasing supply prices of conventional water resources. The water supply sector in ANEMI3 was developed by incorporating water supply as an additional production sector within the newly added energy-economy sector. This has been achieved by adding capital stocks to produce water supply in the form of surface, ground, wastewater reclamation, and desalination water sources. The basic structure of the energy sector, described in the previous section, was adopted as a starting point from which changes were made to accommodate water supply development. The previous versions of ANEMI did not include any distinction between available water resources and water supply for surface and groundwater resources and had no economic component to the development of alternative water resources. In ANEMI3, the addition of the water supply development sector allows for the representation of water supply development from an economic perspective, including both conventional and alternative water supplies.

**Fig 13:**
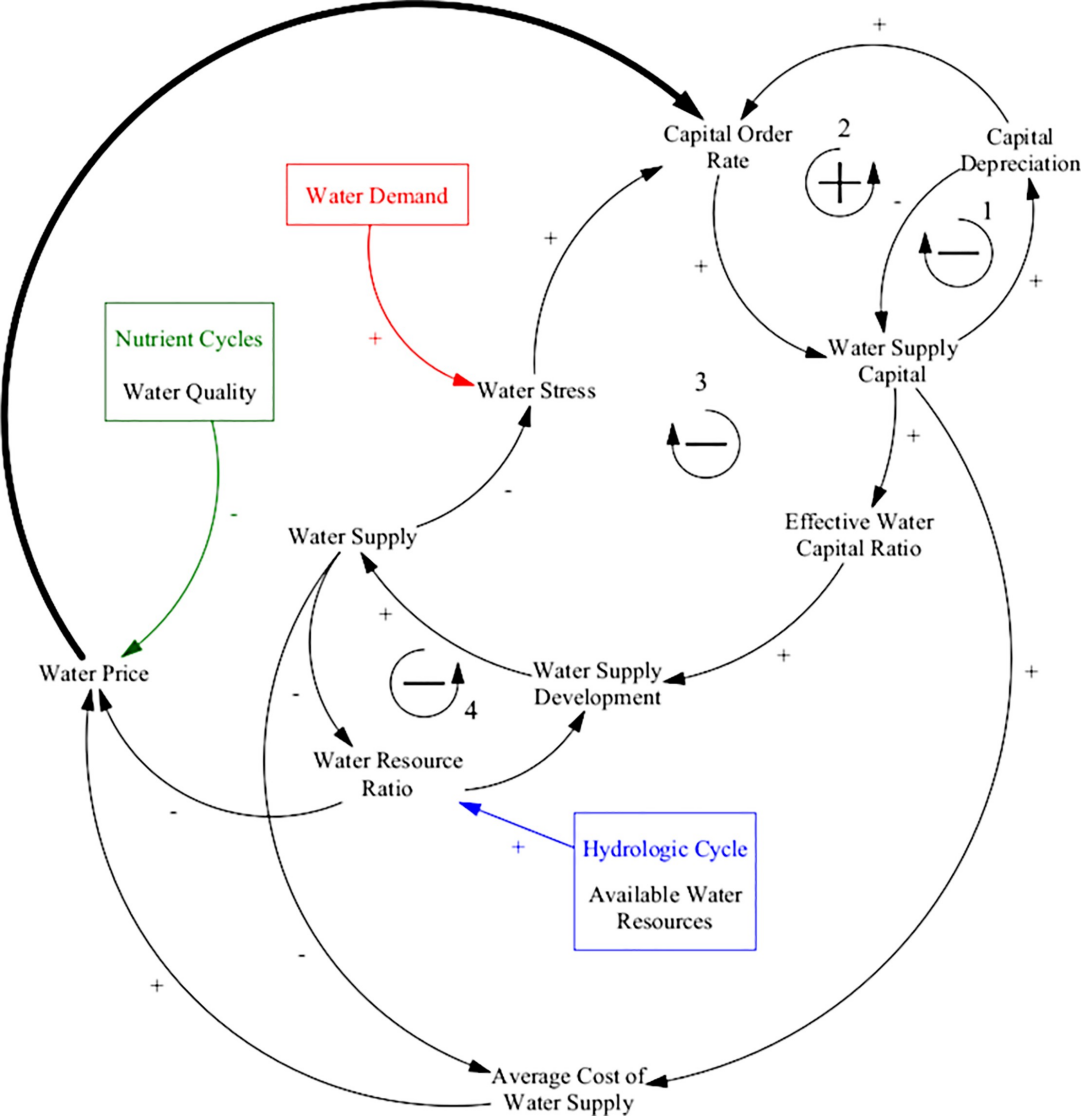
Causal loop diagram of the ANEMI3 water supply development sector. The bolded arrow from water price to water supply indicates a causality that is neither positive nor negative. The entirety of this model sector is a new addition in ANEMI3.

The causal loop diagram presented in Fig 13 illustrates the dynamics that are governing the behaviour of the water supply development sector. The first feedback loop acts as a negative feedback on water supply capital through depreciation. With regards to water supply, this would represent the cost of maintaining supply infrastructure including pumps, distribution networks, dams and reservoirs, and treatment facilities. The second feedback loop counteracts the first, by having a positive feedback effect on water supply capital. With more water supply capital there is more depreciation, which increases the water capital order rate (investment in water supply), thus adding more water supply capital. The third feedback loop is a negative sign that counteracts water stress by prompting investment in water capital to increase water supplies. The fourth and last feedback incorporates the effects of depletion and saturation into water supply development.

As available water resources become depleted, water supply production is reduced for the same input intensity. This means that more effort is required to produce the same rate of water supplies, which also makes a given type of water supply that is depleted more expensive. For example, when the groundwater elevation decreases from over abstractions, more pumping is required to extract the same amount of water resource. The effect of saturation is also included in this relationship, assuming the best or most cost-effective sites are used first for water supply infrastructures. An example of this could include the construction of additional reservoirs, source water intakes, or groundwater wells in areas that are less suitable or cost effective than those that were previously constructed.

The bolded causal link from water price to the capital order rate in Fig 13 indicates a connection that is neither positive nor negative. Instead, this link is used to evaluate the amount of investment that is made in the capital stocks of the different supply types (surface, ground, wastewater reclamation, and desalination water sources). Inputs from the nutrient cycle, hydrologic cycle, and water demand sectors are used to define the water price, water stress, and water resource ratio variables, respectively, in the water supply development sector.

The amount of water resources available for the development of water supplies is dependent on the hydrologic cycle, water demand, and water quality sectors of the model. In the case of surface water, the stable and reusable portion of runoff is taken from the total renewable streamflow and is adjusted for untreated wastewater discharge. The adjustment for wastewater discharge assumes that every cubic meter of contaminated wastewater discharged into water bodies and streams makes unsuitable 8–10 cubic meters of fresh water [31]. The difference in groundwater percolation and discharge is used to consider groundwater resources as this refers to renewable groundwater. Only renewable groundwater resources are considered for the global scale. The inclusion of non-renewable or fossil groundwater resources should be considered at the regional scales. For the potential reuse of wastewater, industrial and domestic wastewaters are considered. Although the reuse of wastewater is highly dependent on the type of wastewater and the use for which it is being treated, it is considered here as a supplementary type of water supply in the case of groundwater and surface water depletion. Water resources used for desalination are considered primarily from the ocean stock in the hydrologic cycle. This results in a virtually limitless supply; however, it is energy-intensive, resulting in a high effective input intensity, thereby limiting production.

The concept of resource depletion in energy production is also applicable to water supply development. For example, in the case of surface water and groundwater resources, depleted water resources will mean less suitable locations for water extraction and treatment plants. This might mean that source waters could be further from where the water is being used, thus increasing distribution costs. Pumping costs could also be increased by using deeper aquifers or surface water supplies that have a more significant difference in elevation from their point of use. Water resource depletion factors into the water supply development process in much the same way as energy production. However, there is one key difference. The depletion effect for energy production is based on the ratio of current energy resources remaining to the initial amount. In contrast, water resources are renewable to varying degrees. Therefore, simply taking the ratio of the available water resources to the initial water resources is insufficient. Here, the ratio of available water resources to the current production level is used.

The concept of endogenous technological change applied to energy production has analogies to water supply development. In the case of surface water and groundwater supplies, it is assumed that pumping, distribution, and treatment technologies will remain largely the same but will show some improvement over time. However, alternative water supplies such as wastewater reuse and desalination are likely to see vast improvements in the near future. Factoring in technological change into the water supply development process is what will help make alternative water supplies more feasible in the future, along with depletion and saturation of conventional water supplies. The dynamics and structure for implementing technological change in water supply development is the same as that of energy technology, however, different parameters are used for desalination and water reclamation technologies and are discussed in [17].

A unique attribute of water resources when considering water supply development is water quality. Degraded water quality can impact the functioning of water treatment facilities as well as maintenance costs and the necessary configuration of unit processes [31–33]. This may also influence the ability to secure adequate source waters for extraction of water resources in the future because of pollution and climate change [32]. This could negatively impact the production of conventional water supplies by increasing the cost of implementing new capital as well as variable inputs needed for treatment and distribution, including energy, chemicals, and labor.

In ANEMI3, nutrient concentrations in surface waters are used as an indicator of water quality on a global scale. Wastewater and agricultural inputs act as the main contributors to water quality degradation through additional nutrient input to surface waters. The amount of total nitrogen and phosphorus-based nutrients in surface water are used as a global indicator of water quality from the nutrient cycle sector of the model.

#### Water quality

The biogeochemical cycle describes the movement of chemical compounds which drive the biological and geological processes that shape the face of the Earth. These compounds move from various reservoirs, including vegetation, soils, rivers and lakes, coastal waters and oceans, and the atmosphere. The processes that drive the movement of these compounds are extremely diverse and occur across widely varied scales of time and space. For example, uplift of the Earth’s crust occurs over millions of years, while the delivery of Nitrogen compounds from the atmosphere to land through lightning strikes can occur in seconds. Some of the most important cycles to consider on a global scale are those associated with Nitrogen (N), and Phosphorous (P). These are some of the main elements that make up living matter and are inextricably linked through the biological processes of respiration and decay [33]. It is not a coincidence that their cycles are also closely tied to human activities and play a vital role for life on Earth in general. The addition of nutrient cycles of nitrogen and phosphorus to the model as indicators of global water quality is a key addition to ANEMI3 compared to the previous versions of the model.

The cycle of N is important to global change research as it has been identified to be an important rate-limiting element with respect to the biological uptake of CO_2_ for land and ocean vegetation, helping to ‘balance the budget’ of carbon through what is known as the ‘fertilization effect’ [34]. Most of the processes included in the nitrogen cycle mirror those of the carbon cycle (although the chemical reactions are different). However, there are a few key differences: the land and ocean plants and organisms also fixate nitrogen from the air in addition to biological uptake; and rain and lightning are important processes for delivering nitrogen from the atmosphere to the Earth’s surface and oceans. Additionally, it should be noted that most of the nitrogen is stored in the air and atmosphere in contrast to carbon where most of it is stored in the ocean.

Phosphorous compounds act as essential nutrients that supports plant life around the globe. The Phosphorous cycle also follows that of the carbon cycle in that the sources and transport processes are similar. The main difference is in the transport of Phosphorous compounds, which occurs through the attachment of sediments that are transported as runoff or in aerosol form. This is partly why the cycle of Phosphorous does not typically include an atmospheric component. Phosphorous rarely exists in a gaseous state unlike nitrogen and carbon but can temporarily form as an aerosol which is deposited relatively quickly. Phosphorous also acts as a rate limiting factor for the biological uptake of carbon and nitrogen, especially for photosynthesizing marine organisms [34].

Humans are now having a profound influence on the major nutrient cycles of N, and P with increasing development and industrialization. N and P are extracted, consumed, and discharged as waste in many cases. This has caused an increase in the amount of these compounds in different components of their respective cycles, thereby accelerating the flow to others. In addition, many of the processes mentioned previously have been bypassed, thus affecting the timing of the cycles themselves. Examples include increasing fertilizer application and soil erosion rates via intensified agriculture, discharging wastewater to streams, and mining P ore for use on land. These human activities have the potential to destabilize the nutrient cycles in ways that have not been seen previously. As a result, we are now able to detect impacts such as climate change, loss of aquatic biodiversity as a result of poor water quality and limited water quantity [35], and acid deposition due to the oxidation of sulfur and nitrogen gases in the atmosphere increasing the pH of rainwater [33]. The extent of these impacts is largely unknown today and less so in the future. However, their potential to impact various aspects of the Earth system, such as population, economy, water quality, land cover, food production, and climate, are likely.

The N and P nutrient cycle structure is represented using a quasi-steady-state model composed of nutrient sources and sinks for the atmosphere, land, humus and soil, rivers, coastal waters, and oceans. The processes used to describe the movement of nutrients within this cycle are based on first-order decay relationships and a series of rate constants. The model is based closely on the work of [36], however, a surface water element was added with rate constants determined to maintain steady state. This part of the model assumes an initial steady state condition from which the model is perturbed to account for human influence on the nutrient cycles. Nutrient inputs represent human influence on the surface water element, including domestic and industrial wastewater and agricultural runoff. This model is used as the basis for the development of nutrient cycles in ANEMI3 [37]. Surface water nutrient concentrations are calculated using the surface water volume of the hydrologic cycle model sector to influence water supply development.

#### Persistent pollution

**Fig 14:**
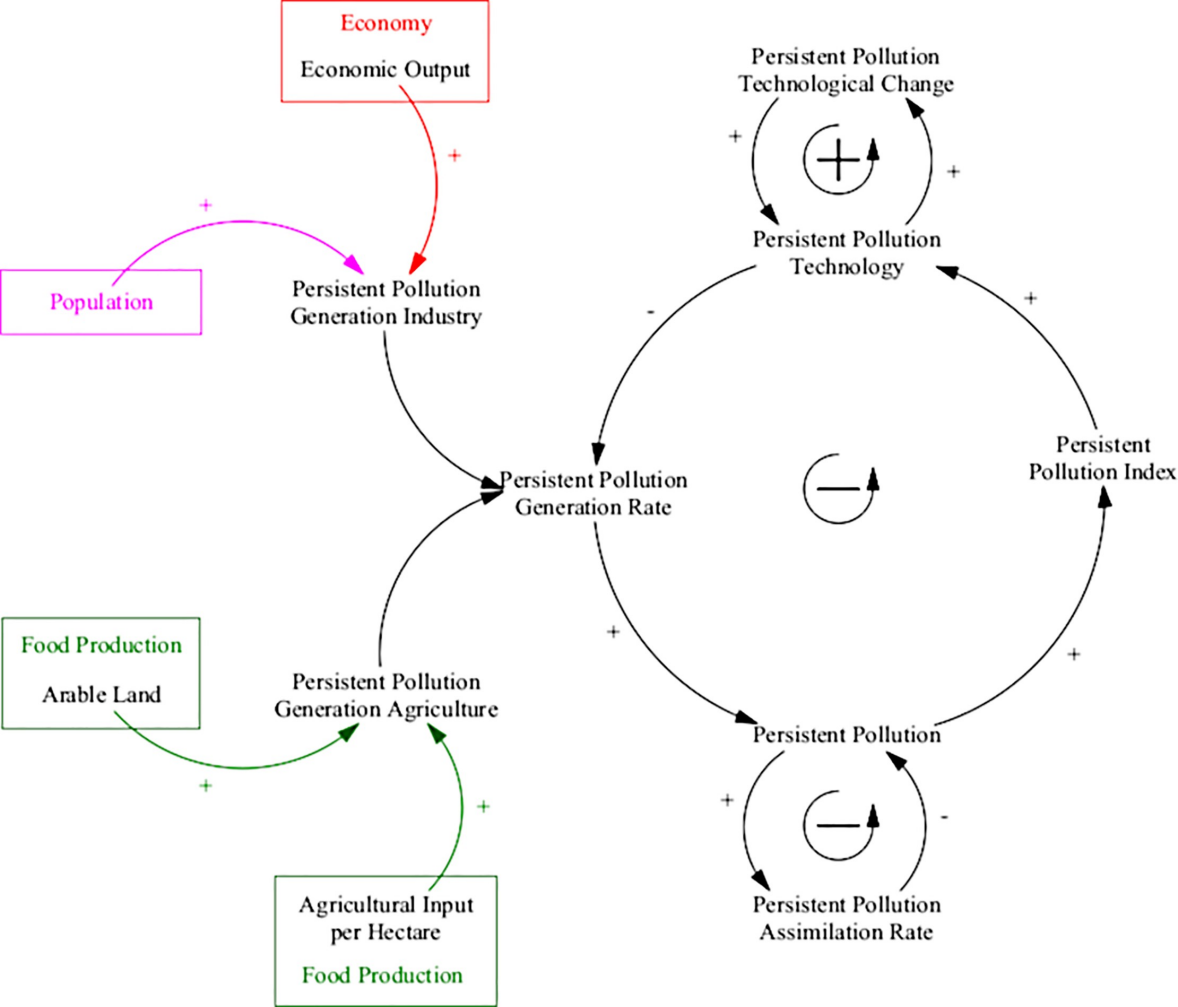
Causal structure of the ANEMI3 persistent pollution sector. The entirety of this model sector is a new addition in ANEMI3.

An additional sector to represent the level of persistent pollution in the Earth system was added in ANEMI3. This sector is used to describe the generation and assimilation of pollutants over time that may be harmful to the global biosphere [38]. It is based on the persistent pollution sector of the WORLD3 model and is used to form additional negative feedback on population growth [23]. The main drivers for the generation of persistent pollution are industrial and agricultural activity, while the current population and economic output are used to scale these effects in the global system. Technological change acts as a reduction factor for the levels of persistent pollution generation from these activities, while the natural rate of assimilation represents the environmental capacity to cope with and break down these pollutants over time. The causal structure of the persistent pollution sector is shown in Fig 14.

There are three feedback loops that drive the dynamics of persistent pollution. The loop connecting persistent pollution with persistent pollution technology acts as negative feedback on persistent pollution. As the levels of persistent pollution increase, so too does the persistent pollution index, creating a greater need for technological change for dealing with pollution. The changes in technology reduce the generation rate from industry and agriculture, which results in less persistent pollution. The positive loop driving technological change represents an accumulation of knowledge, whereby more technological progress leads to a faster accumulation of new developments in persistent pollution technology. The final loop represents negative feedback on persistent pollution through the natural assimilation rate. Over time, assimilation leads to a decrease in persistent pollution, acting as a form of exponential decay.

### Model computation

The ANEMI3 model is parameterized to simulate the Earth system over the period of 1980 to 2100 with an integration time step of 1/128^th^ of year. Due to the rigid nature of the system equations, the simple Euler integration method is used. The model has been parameterized to capture the initial conditions in 1980 and reproduce the behaviour of key system variables in each sector. Details on the parameter estimation, model testing and sensitivity analysis, and comparison to ANEMI2 are provided in the following sub-sections.

### Parameter estimation

Due to the large number of feedbacks in the ANEMI3 model, any changes made in one sector affects all others. This is also true when incorporating and coupling new sectors into the model as additional feedbacks are formed. In order to ensure that realistic values and system behaviours are generated, some of the parameters needed to be re-estimated, while many of the parameter values used in ANEMI2 remain unchanged. Parameters within the water supply development sector and the energy production sector were estimated as they are newly added sectors in the model largely influence the other sectors. The population sector also contained parameters relating to life expectancy and fertility that needed to be re-estimated so that more realistic population values could be obtained, as population growth is a key driver for every sector of the model. Water demand data are used to facilitate parameter re-estimation, as the new economic and energy sector now plays a role in the evaluation of domestic and industrial water demands.

The procedure for selecting the estimated model values is based on an optimization procedure that minimizes errors in global historical datasets for population, water supply, energy production, and water demands. The objective function is extremely non-linear due to the coupled non-linear nature of the model. Modifying any of the decision variables will affect all other aspects of the model to some degree. The solution space is assumed to be one that has many valleys and peaks, creating the potential for finding suboptimal solutions. Because of this, a global optimization algorithm needs to be used rather than a gradient-based method. The differential evolution algorithm [39] was selected for this reason, in addition to the fact that derivatives are not needed for the objective function. This algorithm is evolutionary and stochastic by nature, which can lead to results that are close to the global optimum but not necessarily exact. The minimum solution obtained by the differential evolution algorithm was used as a starting point for a deterministic local minimizer to finish the optimization.

### Model testing

System dynamics simulation models can be constructed to represent purely physical systems for which an input can be given to generate an output that can be compared to data in the real world or analytical solutions of the system. However, this modelling approach is often used to analyze all types of systems that could include social elements or decision-making processes that can be more abstract or where a high degree of uncertainty exists in measurements. That is why in the field of system dynamics simulation true validation and verification are deemed impossible.

A series of tests are used to evaluate the ANEMI3 plausibility of the baseline scenario with regards to the dynamics that take place [16]. The absolute values are important, however the emphasis here is on the model behaviour so that the feedback mechanisms that are driving the model to future states can be analyzed.

Many of the variables in ANEMI3 do not have historically observed counterparts on a global scale, but there are key variables in each sector that can be compared to historical data. One thing to note in this comparison is that on a global scale, there are many datasets that are incomplete (data is only recorded for certain regions), inconsistent (different recording methodologies used across regions, recording is done at irregular intervals), and at times, unreliable. However, there is still value in comparing the model to the real world in any way possible to see that it reproduces the behaviour of the sub-systems that are being represented. With this being said, the goal is not to reproduce the numbers from the data but build confidence in the model’s ability to generate realistic system behaviours so that future behaviours, as well as policies that are implemented to alter them, can be used in decision making. The ANEMI3 variables that have been selected, along with the datasets used for comparison, are in Table 2. It should be noted that due to limited availability of global datasets mentioned previously, the ANEMI3 model has also been parameterized using the datasets mentioned in Table 2 to achieve realistic system behaviours.

**Table 2:** Global datasets used for comparison to ANEMI3 modelled values.

Model Sector	Variable	Datasets
Population	Total Population	[40]
	Population (0–14)	
	Population (15–44)	
	Population (45–65)	
	Population (65+)	
Climate	Global Atmospheric Temperature	[41]
Water Demand	Domestic Water Withdrawal	[42]
	Industrial Water Withdrawal	
	Agricultural Water Withdrawal	
Water Supply	Surface Water Withdrawal	
	Ground Water Withdrawal	
Energy Production	Coal Energy Production	[43, 44]
	Oil and Gas Energy Production	
	Hydro and Nuclear Energy Production	
	Renewable Energies	[44]
Land Use and Cover	Agricultural Area	[45]
	Urban Area	

**Fig 15:**
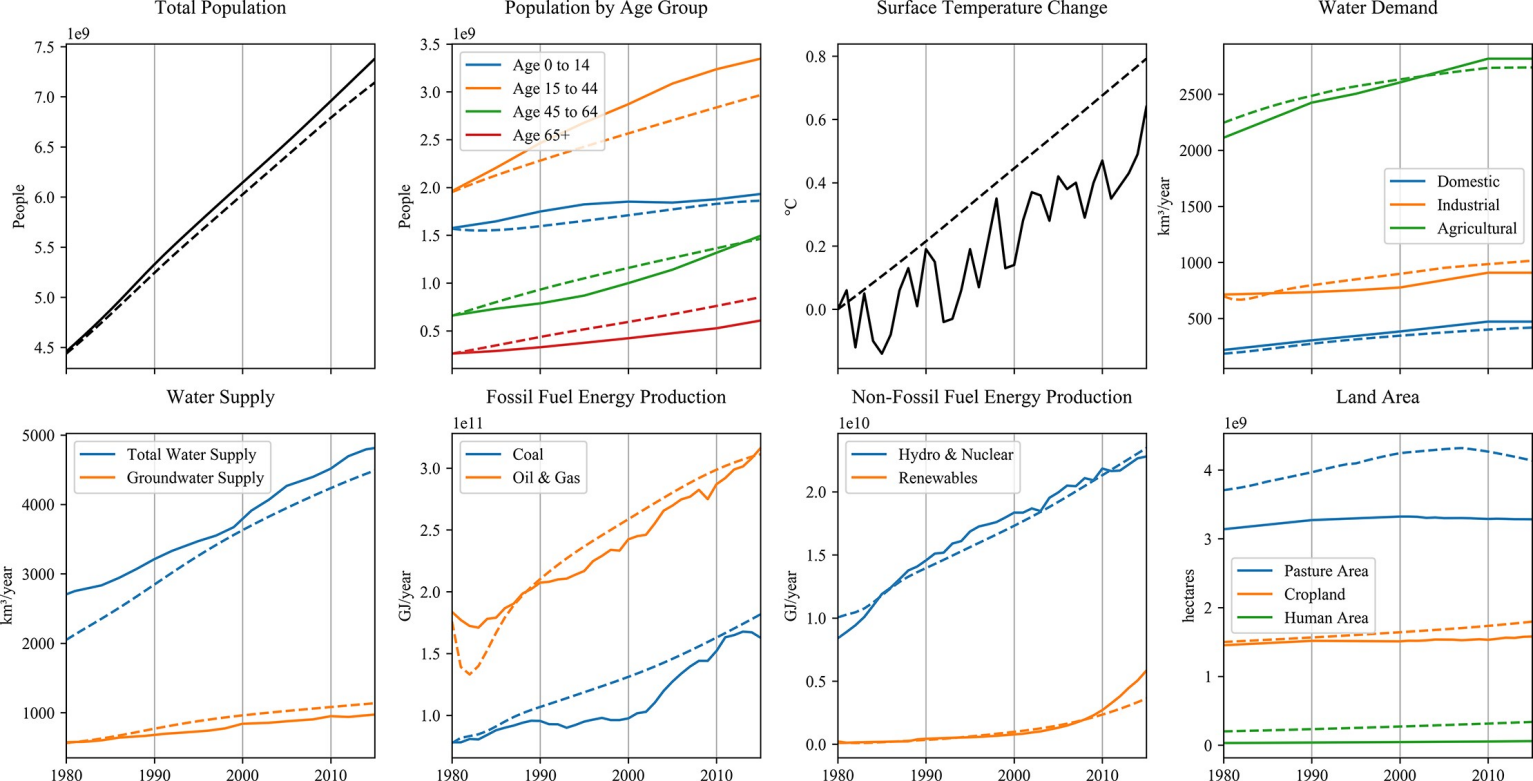
Comparison of modelled ANEMI3 variables to global estimates from the literature. Dotted lines indicated modelled values.

The model variables in ANEMI3 are compared to the global datasets of Table 2 in Fig 15. Overall, the ANEMI3 model captures historical trends, however there are important points to note in this comparison. The comparison of observed surface temperature change shows that ANEMI3 captures long term increase in surface temperature, but not the interannual variation. This is also the case for energy production from fossil fuel and non-fossil fuel energy sources. This is because the ANEMI3 model is made up of relatively simple disciplinary models, which are designed to capture long-term dynamics in each model sector and are unable to capture processes occurring on finer timescales. This could be considered a drawback of the model; however, the model’s main purpose is to further the understanding of global change dynamics. Therefore, simplicity in the disciplinary models that compose the ANEMI3 model is a strength, as it allows for dynamics of the Earth system to be more easily examined.

### Sensitivity analysis

In system dynamics simulation, model uncertainty is exhibited in many forms. This includes the parameters defining model constants, initial values, and the structure of the model itself [46]. Often, this uncertainty is addressed through sensitivity analyses to assess the impact it may have on overall system behavior. Sensitivity analyses can be used to assess various types of uncertainty in system dynamics models including numerical sensitivity, behaviour mode sensitivity, and policy sensitivity [16].

Here, a set of variables were selected from the model to test the sensitivity of the main state variables shown in Table 3. The selected parameters are chosen due to uncertainty in their values or the model structure for which they are used. This will determine whether alternate types of model behaviour are possible by varying the assumed values of these parameters.

**Table 3:** Parameters used to test the sensitivity of key state variables in the ANEMI3.

State Variable	Parameters	Minimum Change	Maximum Change
Population	Water Stress Effects	-10%	+10%
	GDP Effects	-10%	+10%
	Food Production Effects	-10%	+10%
	Pollution Effects	-10%	+10%
Surface Temperature Change	Climate Feedback Parameter	-10%	+10%
	Base Precipitation Multiplier	-20%	+20%
Water Stress	Stable and Usable Runoff Percentage	-20%	+20%
	Wastewater Pollution Factor	-20%	+20%
	Energy Withdrawal Factors	-10%	+10%
	Specific Water Intake Factor for Agriculture	-10%	+10%
	Standard of Living Factor for Domestic Water Demands	-10%	+10%
Food Production	Cropping Intensity of Net Arable Land	-10%	+10%
	Processing Loss Fraction	-20%	+20%
	Average Life of Land	-20%	+20%
	Delay in Cultivation of Potential Arable Land	-20%	+20%
Economic Output	Initial Global Capital Amount	-20%	+20%
Water Supply	Initial Surface Water Supply Capital	-20%	+20%
	Initial Groundwater Supply Capital	-20%	+20%
	Initial Wastewater Reuse Supply Capital	-20%	+20%
	Initial Desalination Supply Capital	-20%	+20%
	Initial Water Producer Prices	-20%	+20%
	Water Supply Construction Delay	-20%	+20%
	Water Elasticity	-10%	+10%
	Water Capital Substitution Elasticity	-10%	+10%
	Water Order Adjustment Coefficient	-10%	+10%
	Attractiveness Width	-10%	+10%
	Water Quality Share Parameter	-10%	+10%
Nutrient Surface Water Concentration	Phosphorus Removal Efficiency from Wastewater	-10%	+10%
	Nitrogen Removal Efficiency from Wastewater	-10%	+10%
	Phosphorus Leaching from Cropland	-20%	+20%
	Nitrogen Leaching from Cropland	-20%	+20%
	Phosphorus Wastewater Concentration	-20%	+20%
	Nitrogen Wastewater Concentration	-20%	+20%
Persistent Pollution	Pollution Assimilation Half-Life	-20%	+20%
	Persistent Pollution Transmission Delay	-20%	+20%
	Technology Development Delay	-20%	+20%
	Industrial Material Toxicity Index	-20%	+20%
	Agricultural Material Toxicity Index	-20%	+20%

Monte Carlo analysis provides an efficient means to test the model sensitivity. Triangular probability distributions were assigned to the selected parameters, with the highest point of probability in the triangle being assigned the baseline value of these parameters. The outer limits defined by the minimum and maximum percentage change to the baseline value. The maximum and minimum change assigned to each parameter for the sensitivity analysis is set as either 10% or 20% based on the assumed level of uncertainty. The model is then run 200 times using input values sampled from these probability distributions, allowing for the distribution of the model output to be examined.

**Fig 16:**
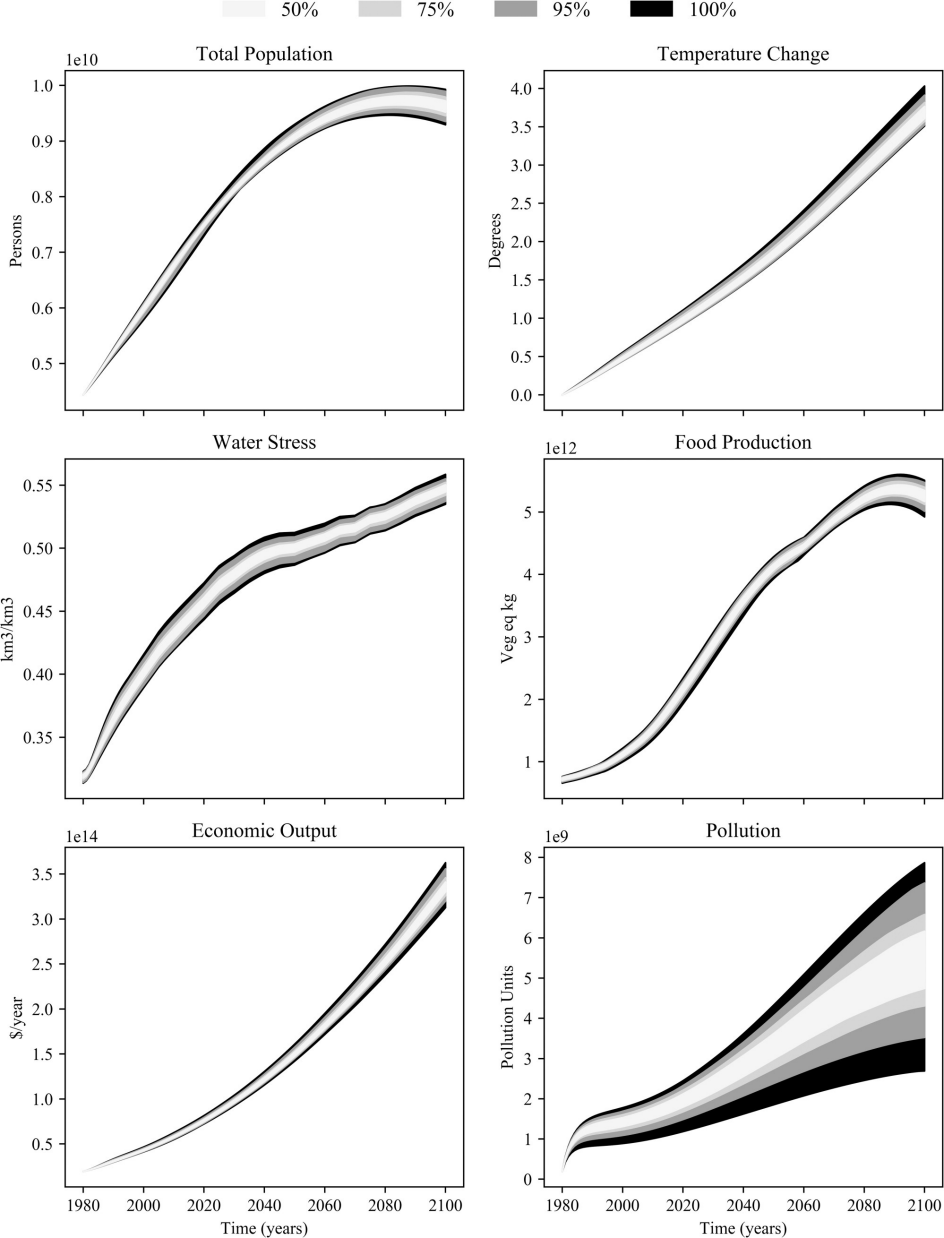
Distribution of select state variables from Monte Carlo sensitivity simulation. Shaded areas denote probabilistic range out state variables based on the input parameters.

The distribution of the state variable outputs from this analysis is shown in Fig 16. By 2100, the global population is shown to range between 9.3 to 9.9 billion, with a global surface temperature change between 3.5 and 4°C, and gross economic output of 312 to 363 trillion 1980 USD. The largest relative range of the set of state variables is shown for the persistent pollution state variable. This indicates that the uncertainty associated with the parameters in the persistent pollution sector can result in a large variation of persistent pollution levels.

Further investigation on the individual affect of the persistent pollution sector parameters was conducted by varying each parameter independently (Table 4). The parameters which have the most impact on persistent pollution by 2100 are shown to be the industrial material toxicity index and pollution assimilation half-life, which can cause persistent pollution to vary by -35% to 49% and -38% to 51% by 2100, respectively. Both parameters do not have real-world analogues to compare to on a globally aggregated scale, and the associated uncertainty can not be reduced. However, the sensitivity analysis has shown that despite the uncertainty in input parameters, the overall behaviour and trajectory of the model state variables remains unchanged.

**Table 4:** Parameters used to test the sensitivity of key state variables in the ANEMI3.

Parameter	Change	Percentage Difference by 2100
Agricultural Material Toxicity Index	-20%	-4%
	20%	5%
Industrial Material Toxicity Index	-20%	-35%
	20%	49%
Technology Development Delay	-20%	0%
	20%	0%
Persistent Pollution Transmission Delay	-20%	2%
	20%	-2%
Pollution Assimilation Half Life	-20%	-38%
	20%	51%

### Comparison of ANEMI2 and ANEMI3

The ANEMI3 model builds from its previous version, ANEMI2 [12]. The significant structural modifications of the model include: (i) implementation of the energy-economy system based on the principles of system dynamics simulation; (ii) incorporation of water supply as an additional sector in the global economy that parallels the production of energy; (iii) inclusion of climate change effects on land yield and potentially arable land for food production, and (iv) addition of nitrogen and phosphorus based nutrient cycles as indicators of global water quality, which affect the development of surface water supplies. Additional modifications to the food production sector are included, as well as the incorporation of a persistent pollution sector which acts to limit population growth and food production. The new features and considerations made in ANEMI3 are documented in Table 5, with further discussion below.

**Table 5:** Major features implemented in ANEMI3 versus ANEMI2.

Model Sector	Feature	Justification
Energy Economy	• Switch to feedback-based disequilibrium model from market clearing partial equilibrium model	• Allows for water supply development sector to be incorporated into energy-economy sector
Water Supply Development	• Inclusion of water supply development as a new production sector in the economic model	• Creates a distinction between available water resources and water supply
		• Links economy to the development of conventional and alternative water supplies
	• Water supply development mirrors energy production structure	
		• Allows for development pathways of water supply types to be examined
Food Production	• Effect of climate change on land yield	• Incorporates effects of heat stress from increasing temperatures on major crop types
		• Limiting effect on food production
	• Effect of climate change on potentially arable land	• Allows for northward expansion of agricultural areas due to climate change
		• Alleviates limitations of arable land on food production over time
Persistent Pollution	• Addition of persistent pollution sector from WORLD3 [20]	• Allows for pollution generation and assimilation to be represented at a global scale
		• It affects food production and population through life expectancy
Water Demand	• Industrial water demand incorporates technological change	• Allows for more realistic water demand assessment from projected changes in energy production technologies

The new energy-economy sector in the ANEMI3 model is based on previous works which have established feedback-based models that bring together energy production and economic development [26–28]. The ANEMI3 model structure of the energy-economy sector, as discussed earlier, is using a completely different approach from the ANEMI2. The implementation is based on the principles of system dynamics simulation. Many of the dynamics related to economic growth and resource depletion from the previous approach are captured. However, the macroeconomic assumption of market equilibrium that is used in ANEMI2 is no longer present. The ANEMI3 approach is based on a disequilibrium model. Instead of energy prices being set to equate supply and demand at every time step, there are negative feedbacks that constantly drive supply to meet the demand as they change over time.

In ANEMI3, water supply was to be added as an additional service to be sold to the firm, and the firm would seek to minimize the total cost of production by considering the prices of supplying water. This is based on the current level of capital stocks in water supply infrastructure for surface water, groundwater, wastewater reuse, and desalination water supplies. The capital stocks include for example, infrastructure such as reservoirs, treatment plants, and distribution networks, in the case of surface water supplies. Connections between energy and water production were to be incorporated into the model by including energy as a key component in the production of water and vice-versa, forming a nexus between energy and water production in the global economy. However, the implementation of this structure into the energy-economy sector of ANEMI2 was not possible as the clearing of the energy and water markets had a very narrow pathway and was extremely unstable. Therefore, in ANEMI3 a new energy-economy model was incorporated, driven solely by feedback processes [27]. Many of the dynamics related to economic growth and resource depletion from the previous approach in ANEMI2 are captured, but there are some key structural differences. The first is that the macroeconomic assumption of market equilibrium used previously is no longer present, as the model being used in ANEMI3 is a disequilibrium model. Instead of energy prices being set to equate supply and demand at every time step, there are negative feedbacks that constantly drive supply to meet the demand as they change over time.

In ANEMI2, water supply was treated the same way as available water resources. Conventional water resources were consumed in response to demand, and alternative water resources increased exogenously in response to water stress. In ANEMI3, water supply infrastructure is developed over time through economic investment for surface water and groundwater resources to be utilized. This distinction allows for alternative water resources to be developed not in response to water stress but to increasing conventional water resources supply prices. This in turn captures the effects of water resource depletion and saturation mentioned previously and allows for an additional factor such as surface water quality to be incorporated through impacts of supply prices. For this reason, a global indicator of surface water quality was needed to represent impacts to water supply. In ANEMI3, this is accomplished by the inclusion of the global nutrient cycles, which represent the natural flow of nitrogen and phosphorus-based compounds and are affected by wastewater discharge and agricultural runoff.

Climate change impacts on food production in ANEMI2 were limited to reductions in arable land due to sea-level rise. In ANEMI3, additional climate change effects are incorporated through impacts of rising temperatures on land yield due to heat stress [24], and potential increase of arable land due to the northward expansion of viable agricultural areas [25].

An additional sector was included to represent persistent pollution from the WORLD3 model [23], limiting both, population growth and food production through life expectancy and land fertility respectively. Industrial water demand in ANEMI3 now incorporates the technological change in energy production by using projected values for different coal, oil and gas, and renewable technologies from the GCAM model [47]. This allows for more plausible industrial water demand projections to be made based on the new energy production structure in ANEMI3.

**Fig 17:**
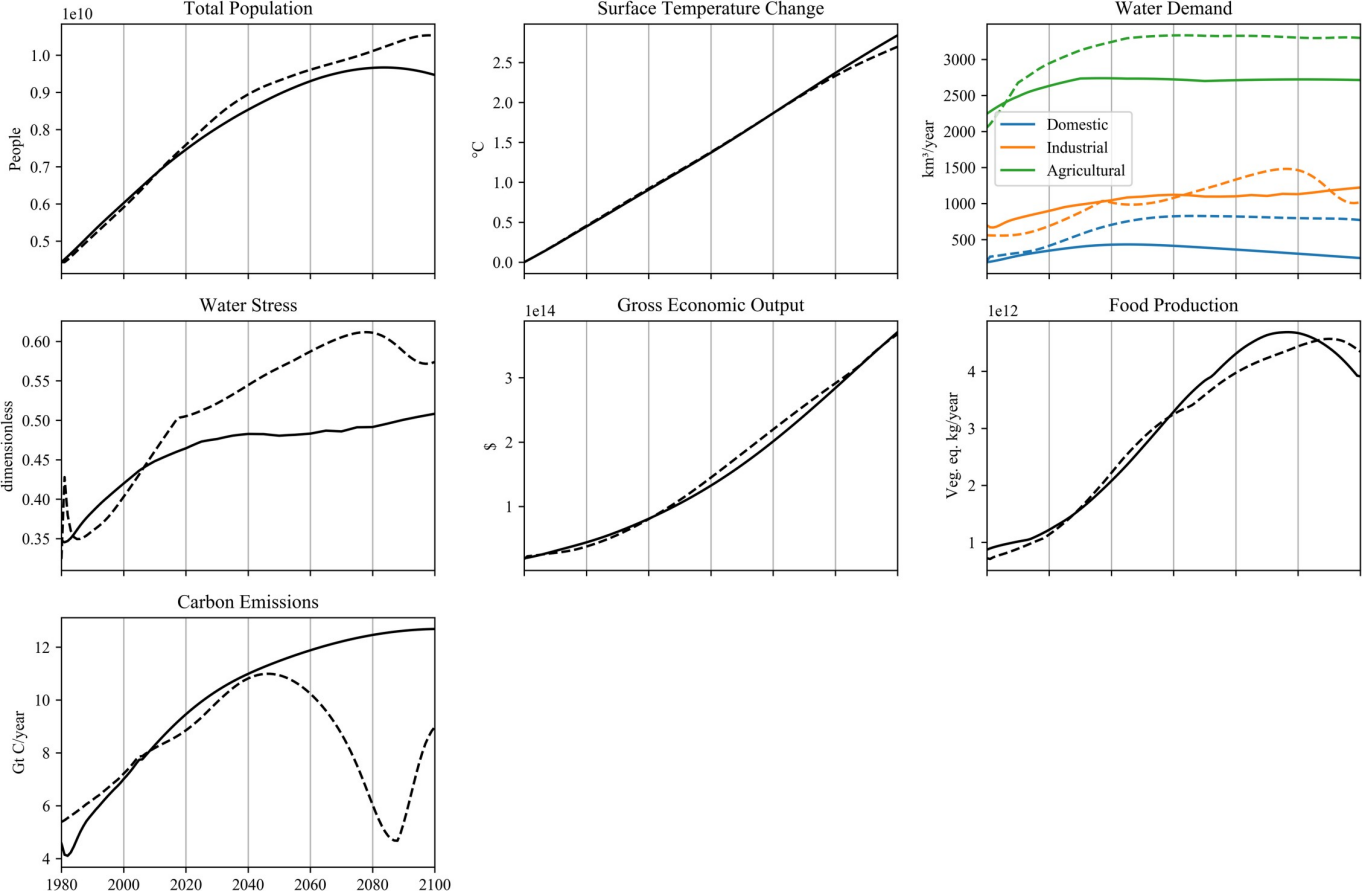
Comparison of simulated values between model variables common to ANEMI2 and ANEMI3. Dotted lines correspond model values for ANEMI2 while solid lines correspond to model values for ANEMI3.

Key model variables in each sector are compared for the results of the baseline run for ANEMI2 and ANEMI3 (Fig 17). In the case of total population and food production, the modelled values are similar between both versions. However, in ANEMI3 the peak total population and food production values is reached slightly sooner due to more negative feedbacks in place. In the case of the total population, this is due to the incorporation of persistent pollution effects on life expectancy, while for food production, this is because of land yield reduction from climate induced heat stress. Carbon emissions vary considerably between model versions in the second half of the 21^st^ century. This is due to the use of a more stable energy-economy model in ANEMI3, which gradually shifts between fossil fuel types (coal, oil, and natural gas) as production prices shift due to depletion of coal and oil reserves. The difference in carbon emissions and their impact on surface temperature between the models is relatively minor, as atmospheric CO_2_ concentrations persist well after emissions occur. Although the energy-economy models are entirely different between ANEMI2 and ANEMI3, the gross economic output follows a very similar trajectory. In the case of water demand, values differ between model versions due to changes in gross economic output per capita, and the new industrial water demand structure that incorporates technological changes. Water stress in ANEMI3 takes a different pathway from ANEMI2 due to the alternative formulation of water stress, which is redefined as the ratio of water withdrawals to the developed water supply. Both formulations result in a value of water stress less than 1, indicating low water stress on a globally aggregated scale. However, the overall trajectory is the same with increasing water stress to the end of the 21^st^ century.

## Model usage

The generalized, feedback-based structure of the ANEMI model allows the user to test policy scenarios related to global change. By altering one of the model parameters at a given point in time, all model sectors will respond through the driving feedback processes which govern the model structure. The output of the model using the baseline parameter set provides a reference scenario to which alternative policy scenarios may be compared. Different parameter sets have the potential to cause a shift in model behaviour compared to the reference scenario, as the dominance of feedback processes in the reference scenario may change. The large number of variables and sectors in the ANEMI3 model allows for the development of a wide variety of scenarios, each with a different focus.

- Thematic scenarios can be developed which target a given theme of global change within the Earth system. For example, suppose the selected theme is focused on climate change mitigation. In that case, policies may be put in place to reduce emissions through mechanisms such as carbon taxation, the substitution of fossil fuels, and land use management. Another theme may focus on the examination of future water stress by altering population growth levels, water availability and demand, and the use of alternative water supplies. Examination of thematic scenario runs can facilitate the development of integrated policy options, which target changes in multiple model sectors to address system behaviour associated with a given theme.
- Scenarios may also be developed to examine more detailed changes to a given sector on the rest of the model. This approach can provide global change context to disciplinary studies that only focus on a given sector’s details. An example of this might include altering rates of assumed technological change effects for alternative water supplies as an adaptation measure for increased water stress in the future. This would represent faster increase in the efficiency and feasibility of alternative water supplies including water reclamation and desalination. Another example may include examining the effects of a new technology for energy production on greenhouse gas emissions and climate change.
- Sensitivity scenarios can be developed to explore the impact of assumed parameter values on Earth system behaviour. By altering parameter values through methods such as Monte Carlo simulation, the relative importance of parameter values can be assessed. Suppose a parameter is shown to be sensitive to changes. In that case, it may be identified as an area where the model structure can be improved to ensure accurate representation of the driving feedbacks. For example, if model behaviour is sensitive to parameters associated with the land use and land cover sector of the model, then a more detailed representation of land use change (i.e., addition of more land use types or processes affecting change in land use and cover) may be considered.
- One of the benefits of representing global change through Earth system feedbacks is that unexpected system behaviours can be uncovered. Scenarios can be developed to explore the potential for unexpected feedbacks to occur by pushing the boundaries of the model. For example, a longer time horizon may be used to examine the outcome of global changes far into the future compared to the reference run, as small changes occurring in the present day may have larger impacts farther into the future. Another example of this model use may be to introduce shocks to the system as a hypothetical scenario. In the case of a global pandemic for example, this may be simulated at a high level as a drop in economic output and increasing mortality rates. The response of which may generate system behaviours that are entirely different from the reference model run.
- The model in it current form may also be used to test issues related to representation of spatial scale. For example, an increasingly important dynamic that is currently not included in the ANEMI3 model due to limited spatial scale is the migration of human population driven by the climate change. It has been estimated that the number of climate migrants could reach 200 million by the year 2050 as a result of shoreline erosion, coastal flooding, and agricultural displacement [22]. Although the ANEMI3 model cannot represent the scale at which climate impacts occur to drive migration, functional relationships may be developed to represent the aggregated effect at the global scale.

## Limitations

The spatial scale used in the ANEMI model is aggregated to the global level. This allows for long-term feedback processes to be examined; however, this level of aggregation limits the level of detail that can be represented. For example, regional processes affecting water stress through variations in water demand and availability cannot be examined without a spatial dimension. Such effects might include regional per capita water usage, population growth rates and migration, as well as regional hydrologic processes used to determine water availability. This could lead to underestimating the impacts of global change on water stress due to only the globally aggregated values being used.

A side effect of the globally aggregated nature of the ANEMI3 model is that processes occurring at finer temporal scales such as climate impacts on flooding and heatwave events cannot be represented even with a finer time step. This is because these events occur on both finer temporal and spatial scales, which the structure of the model cannot represent. For local and regional events to be captured, a downscaling approach would be needed to transform global drivers to local and regional scales. This approach is analogous to the dynamic downscaling of global climate models through a regional climate model which captures local topographic features and surface characteristics.

## Conclusions

This work has documented the ANEMI3 model as a new tool for global change analysis. The feedback-based structure is designed to promote understanding of the feedbacks that drive Earth system behaviour and the process of global change occurring within it. The model sectors of climate, carbon cycle, population, land use, food production, hydrologic cycle, energy economy, water demand and supply, water quality, and persistent pollution are used to represent these feedbacks from a high level. The aggregated spatial scale of the model allows for examining global scale feedbacks through the development of scenarios that focus on individual or multiple model sectors. However, this can also be a limitation as more detail studies at regional and local levels cannot readily be performed. Future work may focus on the development of a downscaling tool to disaggregate the global scale model results to regional scales.
